# Optimization of Moist and Oven-Dried Bacterial Cellulose Production for Functional Properties

**DOI:** 10.3390/polym13132088

**Published:** 2021-06-24

**Authors:** Ioana M. Bodea, Florin I. Beteg, Carmen R. Pop, Adriana P. David, Mircea Cristian Dudescu, Cristian Vilău, Andreea Stănilă, Ancuța M. Rotar, Giorgiana M. Cătunescu

**Affiliations:** 1 Department of Preclinical and Clinical Sciences, Faculty of Veterinary Medicine, University of Agricultural Sciences and Veterinary Medicine Cluj-Napoca, 400372 Cluj-Napoca, Romania; ioana.bodea@usamvcluj.ro (I.M.B.); florin.beteg@usamvcluj.ro (F.I.B.); 2 Department of Food Science, Faculty of Food Science and Technology, University of Agricultural Sciences and Veterinary Medicine Cluj-Napoca, 400372 Cluj-Napoca, Romania; andreea.stanila@usamvcluj.ro (A.S.); anca.rotar@usamvcluj.ro (A.M.R.); 3 Department of Technical and Soil Sciences, Faculty of Agriculture, University of Agricultural Science and Veterinary Medicine Cluj-Napoca, 400372 Cluj-Napoca, Romania; adriana.david@usamvcluj.ro; 4 Department of Mechanical Engineering, Technical University of Cluj-Napoca, 400114 Cluj-Napoca, Romania; mircea.dudescu@rezi.utcluj.ro (M.C.D.); cristian.vilau@tcm.utcluj.ro (C.V.)

**Keywords:** drug-release, SEM, swelling ratio, thickness, TEM

## Abstract

Bacterial cellulose (BC) is a natural polymer with properties suitable for tissue engineering and possible applications in scaffold production. However, current procedures have limitations in obtaining BC pellicles with the desired structural, physical, and mechanical properties. Thus, this study analyzed the optimal culture conditions of BC membranes and two types of processing: draining and oven-drying. The aim was to obtain BC membranes with properties suitable for a wound dressing material. Two studies were carried out. In the preliminary study, the medium (100 mL) was inoculated with varying volumes (1, 2, 3, 4, and 5 mL) and incubated statically for different periods (3, 6, 9, 12, and 18 days), using a full factorial experimental design. Thickness, uniformity, weight, and yield were evaluated. In the optimization study, a Box–Behnken design was used. Two independent variables were used: inoculum volume (X_1_: 1, 3, and 5 mL) and fermentation period (X_2_: 6, 12, and 18 d) to determine the target response variables: thickness, swelling ratio, drug release, fiber diameter, tensile strength, and Young’s modulus for both dry and moist BC membranes. The mathematical modelling of the effect of the two independent variables was performed by response surface methodology (RSM). The obtained models were validated with new experimental values and confirmed for all tested properties, except Young’s modulus of oven-dried BC. Thus, the optimal properties in terms of a scaffold material of the moist BC were obtained with an inoculum volume of 5% (*v*/*v*) and 16 d of fermentation. While, for the oven-dried membranes, optimal properties were obtained with a 4% (*v*/*v*) and 14 d of fermentation.

## 1. Introduction

Bacterial cellulose (BC) has an array of physical, mechanical, and biological properties, such as swelling behavior [1,2], high tensile strength [1,3], high water-holding capability [2], high porosity [4], ultra-fine fiber network [1], biodegradability, non-toxic nature, and biocompatibility, which makes it a promising material in tissue engineering [5,6,7,8]. It is produced by several bacteria as a primary metabolic product in the form of swollen membranes in static culture [5], but also as irregular shape sphere-like cellulose suspensions, in agitated/shaking and bioreactor cultures [9,10]. However, the most effective is *Komagataeibacter xylinus*, formerly known as *Gluconacetobacter xylinum*. It is currently used to produce commercially available BC because of its high productivity [10].

BC has been studied rather extensively, mainly in terms of biosynthesis and applications [6,10]. Different methods and systems to produce BC have been analyzed, and many studies have dealt with the production of BC in the last decades [11,12,13,14,15]. Their main aim was to increase BC productivity by obtaining different morphologies, structures, properties, and applications. They usually tried to increase the BC yield by employing experimental designs and statistical methods to optimize the type and proportion of culture media [13,15]. Response surface methodology (RSM) is one of the current approaches of the BC optimization studies, and it is usually applied on a Box–Behnken design (BBD) [11,12,13,14]. In terms of culture, the static method is most frequently used to obtain BC at large scale, because it is a relatively simple technique [10,15]. However, there seems to be a lack of studies that evaluated the simple inoculum volume—fermentation duration relationship in terms of their effect upon the biomedical properties of BC. Thus, it is not yet clear how these basic process parameters influence the uptake ability of BC.

Pristine BC has a unique macromolecular structure consisting of thin subfibrils, which form microfibrils because of strong inter- and intramolecular hydrogen bonding [5]. The usual purification method of the pristine BC pellicles is the treatment with 0.1% NaOH solution for 1 h at 80 °C, while the main processing of the BC is lyophilization [4,16]. However, this processing affects the properties of the native membrane such as: elasticity or uptake capabilities [7,17,18] important characteristics for biomedical applications. Another simple and cheap processing method is oven-drying. It seems to make the membrane compact and brittle [7,17], because the BC structure becomes more rigid and less porous [18]. However, the effect of this processing on the biomedical properties of BC is not fully described as oven-drying of BC is reported sparingly in the scientific literature.

Thus, the aim of the present study is to determine the optimal combination of inoculum volume and fermentation duration to produce BC membranes with desirable morphological, mechanical, and uptake properties for biomedical purposes, in particular for wound dressing scaffold. RSM was chosen to model, analyze, and optimize the properties of BC (the dependent variables), because they are simultaneously influenced by both inoculum volume and fermentation duration (the independent variables). A BBD was chosen as it is currently of great interest, because it generates a smaller number of experiments with reliable results [11]. Additionally, the effect of oven-drying was evaluated against the pristine water-drained BC using the same methodology and properties of biomedical interest. To the best of our knowledge this is the first study that intends to optimize the fermentation (inoculum volume and duration) and oven-drying of pristine BC in terms of biomedical properties by employing mathematical modelling.

## 2. Materials and Methods

### 2.1. Microbial Strain and Chemicals

Microbial strain: *Gluconacetobacter xylinus* (*Komagataeibacter xylinus*) (ATCC^®^ 700178™); chemicals: glucose, yeast extract, CaCO_3_, Agar, NaOH, NaOCl; 1.6% glutaraldehyde; sodium cacodylate trihydrate (C_2_H_12_AsNaO_5_) buffer, osmium tetraoxide 1%, uranyl acetate 2%.

### 2.2. Fermentation Method

*G. xylinus* was cultivated statically at 26 °C on liquid medium containing 50 g/L glucose, 5 g/L yeast extract, and 12.5 g/L CaCO_3_ [19]. The bacterium was preserved on solid medium (50 g/L glucose, 5 g/L yeast extract, 12.5 g/L CaCO_3_, and 15 g/L agar) at 4 °C and recultivated as described, every 2 to 3 weeks.

The lyophilized *G. xylinus* was reactivated by pouring 1.0 mL of liquid medium to rehydrate the pellet, then the suspension was transferred into a 5 mL tube, vortexed, and incubated at 26 °C for 72 h. The bacteria were cultured 3–4 times until a homogeneous cellulose film was observed on the surface of the medium [20]. The suspension was then cultured on sterile solid medium and incubated (DigitHeat 2001245) at 26 °C for 7 days.

The inoculum solution was prepared by aseptically removing 7 to 9 bacterial colonies of the 7-day old *G. xylinus* culture with a cell spreader and transfer them in a 9 mL sterile saline solution tube. Then, the resulting solution was mixed thoroughly for 5 min using a MaxQ 2000 Vortex Mixer. The resulted bacterial cell suspension was adjusted to 1.5 × 10^7^ cells/mL [21] using a spectrophotometer (Shimadzu UV-1900) at 600 nm absorbance and used as inoculum solution.

The fermentation was carried out in sterilized 120 mL square glass bottles containing 100 mL specific liquid medium. For the preliminary study, the medium was inoculated using varying inoculum volumes (1, 2, 3, 4, and 5 mL corresponding to 1, 2, 3, 4, and 5% (*v*/*v*)) and incubated statically at 26 °C for different periods (3, 6, 9, 12, and 18 days). The maximum of 5 mL was chosen, because higher values decreased the cellulose production [22]. A maximum 18-day fermentation was chosen, because, although the reported fermentation period varied from 4 [15] to 30 days [23], the usual maxim was between 14 and 21 days [12,15,23], supporting our choice. For the optimization study a Box–Behnken design (BBD) experimental design was used with 3 levels for both the inoculum volume (1, 3, and 5 mL) and fermentation period (6, 12, and 18 d) (Table 2). All samples were performed in triplicate.

### 2.3. Purification of Bacterial Cellulose (BC)

BC membranes were collected from the surface of the liquid medium and washed repeatedly (cca. 3 times) with distilled water. Then, BC was treated with 500 mL of 0.1 M NaOH solution for 1 h at 80 °C on a magnetic stirrer to remove all bacteria cells. The BC pellicles (6.5/6.5 mm) were washed again with distilled water and left for 24 h in a 3% NaOCl solution [24]. Afterwards, they were washed with distilled water until reaching a pH of 7. The membranes, thus, obtained were stored either in distilled water at 4 °C until further analysis (thickness, uniformity, weight, water uptake ability, and drug release) or individually vacuum packed in transparent polyethylene foil (for mechanical test, TEM, SEM, and FTIR).

### 2.4. Processing of BC Membranes

Throughout the experiment, 2 types of membrane processing were analyzed: half of the batch was oven-dried (DigitHeat 2001245) at 40 °C until constant weight (cca. 30–60 min), resulting dry BC pellicles (BCd) [8], while the other half was pressed with filter paper until almost all the water in their structure was removed, resulting drained, but moist BC pellicles (BCm).

### 2.5. Preliminary Study

#### 2.5.1. Transmission Electron Microscopy (TEM)

Two types of samples (32.5 × 32.5 mm) were used for TEM analysis: pristine untreated and unpurified BC pellicles and purified BC membranes. TEM was used for the analysis of the cellulose-forming bacteria and evaluation of the purification process. The cellulose films were treated with 1.6% glutaraldehyde (1 h fixation) and buffer (0.1 M, pH 7.4). Each sample was washed 3–5 times every 5–10 min and left in the buffer for 2 days. Then, the samples were fixed for 1 h in osmium tetraoxide 1%, incubated in the dark for 2 h in uranyl acetate 2%, dehydrated in different concentrations of acetone and incorporated into acetone/resin in a RT agitator, then infiltrated in resin Spurr/epon 100% and polymerized. Finally, the samples were cut ultrafine and analyzed with a transmission electron microscope (ZEISS LIBRA 120) at 80 kV. The images were analyzed using ImageJ 1.48 software to assess the morphology of *G. xylinus*.

#### 2.5.2. Film Thickness and Uniformity

The film thickness of BC native purified membranes (65 × 65 mm) was measured using a digital caliper (WIHA 29422). Five measurements were taken for each sample at various random locations. The uniformity was determined as standard deviation (SD) of the 5 measurements. All the measurements were done in triplicate, and the results were expressed as mean ± SD.

#### 2.5.3. Film Weight and Yield

The weight of native BC, BCd, and BCm (32.5 × 32.5 mm) was accurately measured using an analytical balance (KERN ABT 100–5 NM). The dry mass was determined gravimetrically by oven-drying the samples at 40 °C until constant weight (cca. 30–60 min) [18]. Then, the water content and BC yield were computed [25,26]. All the measurements were done in triplicate, and the results were expressed as mean ± SD.

### 2.6. Optimization Study

#### 2.6.1. Experimental Design and Target Optimal Levels for the Response Parameters

A Box–Behnken design (BBD) experimental design [11,12,13,14] was used to optimize the fermentation conditions to obtain BC with properties fit for biomedical purposes: drug release (Y_1_) [4], mechanical properties (Y_2_) [3], structure (Y3) [27], as response variables. Two independent variables were considered at 3 levels: inoculum volume (X_1_) at 1, 3, and 5 mL and fermentation period (X_2_) at 6, 12, and 18 d. The levels were selected based on the results from our preliminary study. The BBD generated 15 experimental runs for the fermentation conditions (represented by X_1_ and X_2_) needed to obtain the target response variables (Y_1_, Y_2_, Y_3_) for the response surface methodology (RSM). Three replicates were set at the center of the design for the estimation of the pure error sum of squares [28,29]. The experimental runs were randomized and carried out in unblocked design (Table 2).

The following levels for each of the response variables were targeted for the multi-response analysis in the optimization procedure: a target thickness of 2 mm [30], highest half-swelling time [31], highest drug half-release time, highest tensile strength, minimum Young’s modulus [1,31,32], and minimum fiber diameter [7].

#### 2.6.2. Fourier Transform Infrared Spectroscopy (FTIR)

The infrared absorption spectrum for each sample was recorded using the Shimadzu IRPrestige-21 spectrophotometer [17,18,24]. Because of the structure of BC, the sample was applied directly to the ATR (attenuated total reflectance) horizontal diamond accessory with a single reflection from PIKE and pressed using its Hight force clamp. The spectrum was recorded at a wavelength range of 600–4000 cm^−1^, with a resolution of 4 cm^−1^ with 16 scans per measurement, according with previous studies [33]. Cellulose from filter paper was used as a reference for the functional groups in BC. The primary data obtained were processed using the IR solution Software programs Overview (Shimadzu) and OriginR 7SR1 Software (OriginLab Corporation, Northampton, USA).

#### 2.6.3. Water Uptake Ability: Swelling Ratio and Moisture Content

The initial weights of BCd and BCm (32.5 × 16.25 mm, prepared as described before) were accurately measured. Then, each sample was immersed in a vial containing 20 mL of deionized water and incubated at room temperature [1]. At precise intervals (10, 20, 30 min; 1, 6, and 24 h), the swollen membranes were weighted after the removal of excess surface water by gently tapping with filter paper. The swelling behavior and moisture content of the membranes were determined using Equations (1) and (2).

Sr (%) = (W_wet_ − W_dry_)/W_dry_ × 100 (1)

Mc (%) = (W_wet_ − W_dry_)/W_wet_ × 100 (2)

where Sr = swelling ratio; Mc = moisture content ratio; W_wet_ = weight of swollen pellicles; W_dry_ = initial weight.

The half-swelling time was obtained by plotting the Sr by time and computing the duration in which the pellicles swell at the half of their W_wet_ [34]. All the measurements and computations were done in triplicate and the results were expressed as mean ± SD.

#### 2.6.4. Drug Release and Drug Half-Release Time

The kinetics of drug release was estimated using beet juice, as immersion fluid [35]. Firstly, a standard curve of beet juice concentration versus absorbance at 520 nm (Shimadzu UV-1900) [35] was obtained by measuring various dilutions (0.1, 0.3, 0.5, 0.7, 1, and 1.5%). The obtained curve had the following equation: y = 0.3644x + 0.0007 and an R^2^ of 0.9996.

For the actual drug release assay, BCd and BCm (32.5 × 16.25 mm) membranes were loaded with concentrated beet juice overnight. The membranes, thus, prepared were immersed in 20 mL distillated water and a 2 mL solution was taken at precise time intervals (30 min; 1, 2, 3, 6, 24, 48, and 72 h), and their absorbance was measured at 520 nm (Shimadzu UV-1900) to determine the beet juice release [35]. After removing aliquots of 2 mL from each sample for analysis, the same volume of fresh distillated water was added [36]. UV absorbance was measured to determine the concentration of released beet juice at each time point [37]. The drug half-release time was obtained by plotting the released beet juice by time and computing the duration in which the pellicles release half of their total uptake [34]. All the measurements and computations were done in triplicate, and the results were expressed as mean ± SD.

#### 2.6.5. Mechanical Properties

The mechanical properties of the BCd and BCm samples were determined by standard tensile tests. All measurements were carried out at room temperature (23 °C) and humidity in the range of 45–50%. Specimens of 6.5 cm in length, 2 cm in width, and 0.6 mm thick were loaded to failure with constant crosshead speed (2 or 4 mm/min) using a tensile test machine (Instron 3366 (10 kN) [38]. Five specimens were tested for each BCm and BCd sample. The maximum load (N), tensile strength (MPa), elongation at break (%), Young’s modulus (MPa), and stiffness (kN/cm) were calculated. The results were reported as mean ± SD of 5 measurements.

#### 2.6.6. Scanning Electron Microscopy (SEM)

Prior to SEM analysis the BCm were treated with 1.6% glutaraldehyde in a sodium cacodylate trihydrate (C_2_H_12_AsNaO_5_) buffer (0.1 M, pH 7.4) for 1 h; afterwards, each sample was washed 3–5 times every 5–10 min with the C_2_H_12_AsNaO_5_ buffer and, then, left in the buffer solution for 1 d. The next day the samples were lyophilized in a critical point drier. BCd and processed BCm membranes were sprayed with Au and Pd (80:20 ratio) in a sputtering apparatus (Leica EM ACE600). All samples were prepared in duplicate and analyzed with ZEISS EVO electronic microscope [16,18]. Image analysis and fiber diameter measurement were performed with ImageJ 1.48 software. The diameters were analyzed in 5 different image fields per each sample measuring the diameter of minimum 100 fibers per filed [16].

#### 2.6.7. Statistical Analysis, Response Surface Methodology (RSM), and Model Fitting

XLSTAT (version 2020.4.1) and Minitab^®^ (version 19.2020.1) statistical software programs were used to analyze the data. A one-way ANOVA (*p* < 0.05) within samples was used to compare the effects of inoculum, harvest day, and processing type. Fisher pairwise comparisons (LSD, *p* = 0.05) was applied whenever ANOVA indicated significant differences among the samples. Pearson correlation coefficients were computed. Linear regression analysis was used to quantify the effect of harvest day, inoculum volume, and processing with a confidence interval of 95%, a tolerance of 0.0001, and a model selection based on best model by adjusted R^2^.

Correlational principal component analysis (PCA) was performed on the preliminary data formatted in observations/variables table [39]. Three of the 5 identified factors (components) were selected: F1, which had an Eigenvalue of 2.67 and accounted for a variability 53.48%; F2, which had an Eigenvalue of 1.11 and accounted for a variability 22.15%; F3, which had an Eigenvalue of 0.72 and accounted for a variability 14.45%.

Response surface methodology (RSM) was used on a fully factorial design with 5 levels for the preliminary study and on a Box–Behnken experimental design for the optimization procedure [11,12,13,14]. For the preliminary study, 2 continuous explanatory variables (X_1_: harvest day, X_2_: inoculum volume) were chosen and 1 response variables variable (Y_1_: thickness). For the optimization study, the 2 continuous explanatory variables (X_1_: harvest day, X_2_: inoculum volume) were kept, and 1 categorical variable was added, X_3_: type of membrane. Their effect was modelled on 6 response variables (Y_1_: thickness; Y_2_: half-swelling time; Y_3_: drug half-release time; Y_4_: tensile strength; Y_5_: Young’s modulus; Y_6_: fiber diameter). Linear, interaction, and squared coefficients of the model were determined by least square regression. A stepwise approach was followed to select the terms in the mathematical model, and an α ≤ 0.15 was set for a term to enter the model. Additional terms were added in the final step to maintain the hierarchical model. Two-dimensional response surface charts and the desirability functions were also computed. ANOVA and Mann–Whitney two-tailed test were performed to analyze the fitting of the model. For response optimization the following goals were set: thickness—target to 2 mm; fiber diameter—minimum; swelling half-life—maximum; drug-release half-life—maximum; tensile strength—maximum; Young’s modulus—minimum. For the validation of the models, new experimental runs were compared with the PI 95% of each theoretical value of the response parameters.

## 3. Results and Discussion

### 3.1. Preliminary Study

#### 3.1.1. Transmission Electron Microscopy TEM

**Figure 1:**
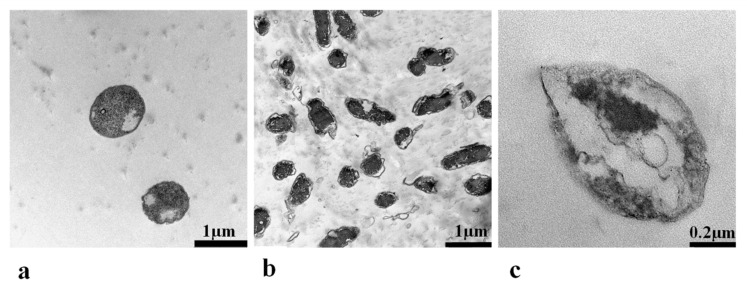
TEM images of *Gluconacetobacter xylinus* cellulose forming bacteria in pristine bacterial cellulose membranes. (**a**)—rod-shaped bacteria; (**b**)—ellipsoidal bacteria; (**c**)—cell structure and wall aspect of *G. xylinus*.

Both purified and unpurified pristine BC membranes were analyzed to assess the effectiveness of NaOH and NaOCl purification treatment. In pristine BC, the bacteria cells occurred randomly in the membrane structure. The bacteria had ellipsoidal to rod shapes with dimensions between 0.3 and 0.7 × 0.8 and 1.4 µm (Figure 1a,b) similar to current reports [31]. The bacteria wall was between 0.02 and 0.03 µm (Figure 1c). This confirmed that the *G. xylinus* cultures were not infected during the handling and fermentation with other species.

Purification is a crucial step in the production of any cellulose product, and more so when aiming a biomedical application. This process is intended to remove all non-cellulose materials such as proteins and nucleic acids derived from bacterial cells and the culture medium. Additionally, the purification process allows the formation of strong inter- and intra-fibrillar hydrogen bonds [24]. The most widely used purification procedure is the treatment of the harvested pellicles with 0.1 M NaOH at 60–80 °C for 1–3 h; then, repeatedly washing with distilled water until they reach a neutral pH [18,24,40]. The proper purification of BC is important, because molds usually start to grow within a few weeks in improperly purified BC membranes, which become darker and opaque [24].

In our case, the pristine BC films were yellow when harvested from the culture medium and became brownish after the NaOH treatment, with random transparent areas. However, after a 24-h NaOCl treatment, the BC membranes reached transparency, showing the desired gel-like structure. TEM imaging confirmed the effectiveness of the treatment, as no rod-shaped bacteria nor other impurities were present in the internal structure of the membrane, even after 6 months. Thus, the two-step treatment that we proposed optimally purified the BC and stopped mold growing. This ensured longer storage, up to 6 months, without experiencing any change in color and/or quality.

#### 3.1.2. Film Thickness, Uniformity

BC samples had different thicknesses according to the inoculum volume and harvest day (Supplementary Materials Table S2). The thickness varied for all pellicles from 1.34 ± 0.20 mm on the 6th harvest day to 2.67 ± 0.67 mm on the 18th day. It was also observed that all samples inoculated with 1 mL were thicker than other samples; however, the difference was significant only on the 9th day of harvest.

The thickness of the films was significantly influenced by both the inoculum volume (*p* < 0.008) and the harvest day (*p* < 0.0001), as resulted from linear regression analysis. However, the duration of fermentation was the most influential factor. Thus, the inoculum volume was removed from the regression analysis, as suggested, and a linear equation was obtained as seen in Equation (3).

Y_1_ = 1.19 + 6.56 ∙ 10^−2^ ∙X_1_ (3)

where Y_1_: thickness (mm), X_1_ = harvest day (d).

**Figure 2:**
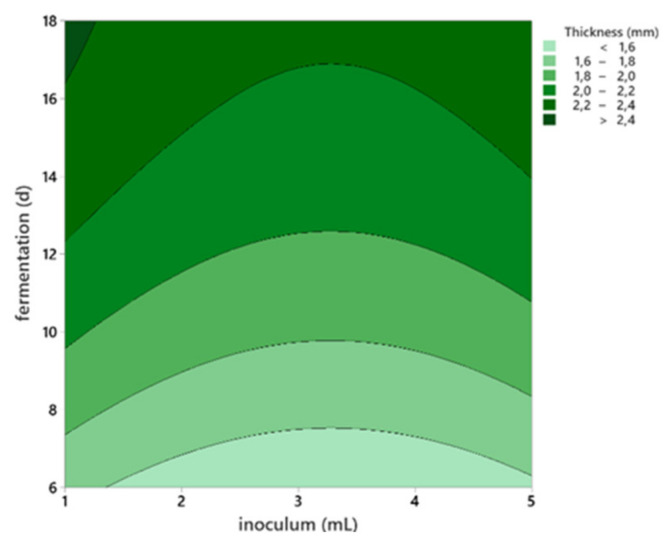
The response surface plot of thickness performed on the factorial design with 5 levels.

Although the model was statistically confirmed, only 47% of the variability was explained by the parameter day (R^2^ = 0.47). Thus, a further response surface regression of thickness as a response variable was performed on the factorial design with five levels (Supplementary Materials Table S2), and the model presented in Table 1 and Figure 2 was generated.

Rangaswamy, Vanitha, and Hungund [22] concluded that the inoculum volume plays an important role in BC production. Similar to our study, the results showed that BC production increases with the inoculum volume. However, this seems to be true until the inoculum volume reaches the threshold of 5% (*v*/*v*). Higher percentages generated a decrease in cellulose production. [22]. Although cellulose production was observed in all inoculum size tested, values lower or higher than 5% inoculum showed a decrease in cellulose production. In contrast, other studies concluded that the total cell count is not significant for BC production [25], but is the number of bacteria within the aerobic zone is, because they are producing the cellulose. Thus, based on our results and previous reports, we kept the inoculum volume in our experimental design for the optimization study.

The uniformity was not influenced by inoculum nor by fermentation period; however, there was a significant difference between the samples (*p* = 0.033), as seen in Supplementary Materials Table S2. Additionally, thickness was negatively correlated with the uniformity (r = −0.595; *p* < 0.05) in Pearson correlation analysis. Thus, the thicker the membrane, the more uniform it tends to be.

Similar to our results, some studies showed that the thickness may vary from 0.01 mm at 48 h incubation period [41] to 8 mm after 2 weeks [42] in a static culture. However, other studies reported no apparent variations or obvious imperfections after direct visual evaluation of BC membranes [43]. Nonetheless, the thickness of BC films may be controlled by adjusting the incubation period and improving the fermentation method [30].

#### 3.1.3. Film Weight and Yield

An increase in both dry weight and water content was observed with the increasing fermentation period (Supplementary Materials Table S2). The dry weight varied from a minimum of 2.63 ± 1.10 to 9.83 ± 1.46 mg, that is approximately from 0.3 to 0.9%, while the yield varied from 0.11 ± 0.04 to 0.39 ± 0.06 g/L. BC yield was the lowest in all 1 mL inoculum samples. The productivity in BC increased from the 6th harvest day (0.11 ± 0.04 g/L) to the 15th day (0.27 ± 0.15 g/L). The 15th fermentation day had the highest cellulose yields, in accord with current studies [25,26]. Hornung, Ludwig, Gerrard, and Schmauder [25] reported that BC yield increased with fermentation time, but cellulose formation ceased, however, after 15 fermentation days. Castro, Zuluaga, Alvarez, Putaux, Caro, Rojas, Mondragon, and Ganan [26] observed that even though BC yield increased from the 2nd to the 8th incubation day, and BC production decreased between the 8th and 14th fermentation day.

Our results are in line with current results, which state that the content of BC in its initial state is no higher than 1% [22,26,44]. BC is a hydrogel-like membrane, and its fibrous and network-like structure contribute to its very high water content [24]. This property is important when considering its biomedical application as wound dressing material [6,7].

The regression analysis showed that the harvest period significantly influenced BC dry weigh (*p* < 0.002), water content (*p* < 0.0001), and yield (*p* = 0.002), while the inoculum volume seemed to have no significant influence.

### 3.2. Optimization Study

For the optimization study, a BBD experimental design was used to obtain the variable combinations for the experimental runs (Table 2). This enabled the experimental runs of a smaller number of samples with reliable results, because the number of levels for the factors is minimized (from five to three in our case) [45,46], and it generates less extreme experimental combinations compared to a central composite design (CCD) [47]. The prediction precision around the supposed optimum is similar to the CCD, because the center point level is repeated (three times in the current study), but with fewer runs [48]. However, the efficiency of BBD was shown to be higher than CCD and three-level full factorial [28,45], providing a model with a better fit. Thus, by using the BBD, the number of experiments was significantly reduced compared to the fully factorial design with five levels as in the preliminary study. In addition, less extreme experimental combinations were tested.

#### 3.2.1. Fourier Transform Infrared Spectroscopy (FTIR)

Fourier Transform infrared spectroscopy (FTIR) analyzes cellulose using the chemical bonding present in the biopolymer. It is an alternative for qualitative analysis, and it enables the identification of BC types and their purity [18,24,49,50].

**Figure 3:**
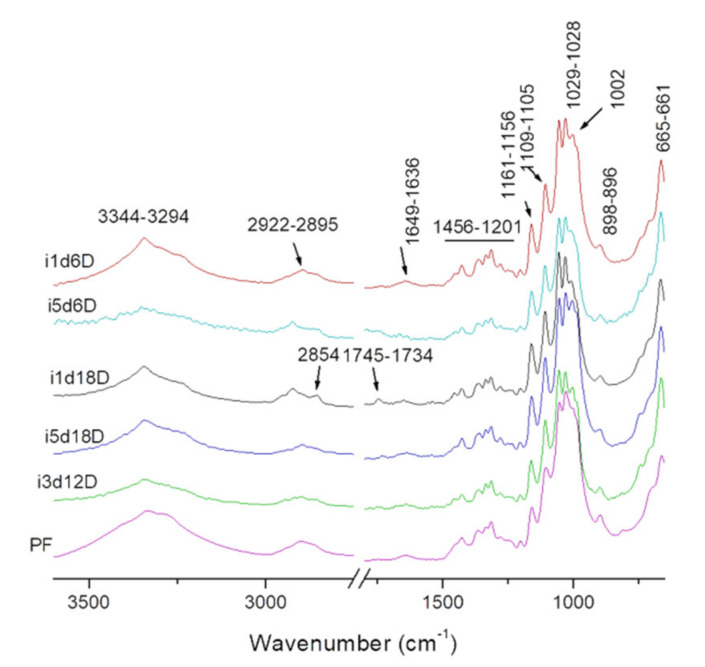
FTIR spectra of bacterial cellulose and filter paper on 650–3600 cm^−1^ domain. PF—filter paper, i—inoculum, d—harvest day, M—moist membrane, D—dry membrane; the number following “i” stands for inoculum volume 1, 3, 5 mL and the number following “d” represents the harvest day 6, 12, 18 days.

In this study, the functional groups of all five variants of BC were confirmed by comparison with the infrared spectrum of filter paper (FP). As shown in Figure 3, the bacteria *G. xylinus* produced BC containing 20 identified functional groups, the majority similar to FP. The adsorption at 3332 cm^−1^ present in FP spectra could be attributed to stretching vibration of intra and inter O-H bond in cellulose [51]. This absorbance peak is present in all BC samples, but its position seemed to vary slightly with the volume on inoculum. The peak was positioned at lower wavenumbers for samples with 5 mL inoculum and higher wavenumbers for samples with 1 mL, probably because of the variations in the BC structure.

The 2899 cm^−1^ peak could be attributed to C-C stretching of CH_2_ and CH_3_ groups or CH_2_ asymmetric stretching [33]. The FP spectra shows a peak at 1647 cm^−1^ corresponding to H-O-H bending of absorbed water [51]. In BC samples, this peak appears shifted to lower wavenumbers: 1645 to 1639 cm^−1^. The peak at 1427 cm^−1^ present in all samples may correspond to CH_2_ scissoring [52], but most studies attribute it to CH_2_ symmetric bonding or O-H in plane bending [53,54]. The peak at 1365 cm^−1^ in FP spectra could be assigned to C-H bending. This peak was shifted to lower wavenumbers in all BC samples, similar to other peaks. The peak at 1334 cm^−1^ could correspond to C-H deformation or O-H in-plane bending [55] and absorption at 1315 cm^−1^ may be assigned to out-of-plane wagging of the CH_2_ groups. The absorption at 1159 cm^−1^ is observed in FP and all BC spectra, with a shift to higher wavenumber (1161 cm^−1^) for samples with 1 mL inoculum. This is a typical indicator of the presence of C-O-C antisymmetric bridge stretching of 1,4-b-O-glucoside in BC [32].

It was shown that the peaks around 1000–1100 cm^−1^ can be assigned to C-O stretching vibrations in primary alcohol and C-O-C skeletal vibrations [54], but this hypothesis is controversial. For example, some studies attributed the absorption at 1030 cm^−1^ and 1054 cm^−1^ to the bending of C-O-H bond of carbohydrates [56,57] or C-O-C pyranose ring skeletal vibration [51,58]. These peaks are also present in all the studied samples. Gao et al. [59] reported that the peak at 1029 cm^−1^ (present in FP and all BC samples spectra) might be also associated with the presence of OCH_3_, while the peak at 1107 cm^−1^ (present in FP and all BC samples spectra) indicated C-C bonds of the monomer units of polysaccharides or C-O bending vibration [55]. An intense peak was observed at 1003 cm^−1^ in the BC spectra, originating from the stretching vibrations of C_3_-O_3_, which was the main bonding forming a cross-linking structure [60].

The peak at 896 cm^−1^ present in FP and all BC samples spectra could be assigned to antisymmetric out-of-phase ring stretching of beta-glycosidic linkages between the glucose units, which is designated as an amorphous absorption band [61,62].

In conclusion, BC produced by *G. xylinus* seems to consist mainly of pure cellulose I because of the two weak peaks at around 1427 and 898 cm^−1^ and other peaks corresponding to pure cellulose [62,63]. Additionally, we found peaks at around 3338, 1160, and 900 cm^−1^, all well studied previously and attributed to cellulose I [64,65]. The peaks observed at 1334, 1315, 1278, and 1427 cm^−1^ show, however, evidence of the presence of cellulose II as well [64,65]. The presence of these peaks in all samples could be attributed to the NaOH treatment used for the purification of BC, which lead to some structure transformation of cellulose type I to cellulose type II. In this case, cellulose II, regenerated cellulose, may have been derived from cellulose I due to the alkali treatment. Similar peaks attributed to the NaOH treatment of BC were previously reported by several authors [66,67].

#### 3.2.2. Water Uptake Ability: Swelling Ratio and Moisture Content

**Figure 4:**
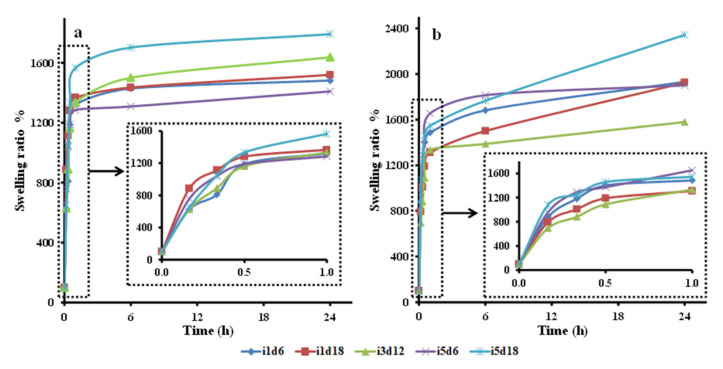
Swelling ratio of dry and moist bacterial cellulose (BC) membranes over 24 h. (**a**)—moist BC membrane; (**b**)—dry BC membrane; i—inoculum, d—harvest day; the number following “i” stands for inoculum volume 1, 3, 5 mL and the number following “d” represents the harvest day 6, 12, 18 days.

The BCd and BCm batches took up significant amounts of water during the 24 h (Figure 4, Supplementary Materials Table S3), both showing significant different swelling ratios at the end compared with the beginning of the trials (*p* < 0.0001, *p* = 0.0004, respectively). The swelling activity among BCd samples was statistically different only up to the first 10 min (*p* = 0.046). The samples obtained with 5 mL inoculum and harvested after 18 d had the highest swelling ratio after 10 min (1079.41 ± 81.60%) (Figure 4b), while the membrane with 3 mL harvested after 12 d, which had the lowest (694.79 ± 66.50%). The swelling activity up to 10 min was significantly influenced by the volume of inoculum used to obtain the BC (*p* = 0.039), while the harvest day seemed to have no significant individual influence. Li, Jiang, Zheng, Gong, Chen, Jiang, and Yang [3] showed that the water uptake ability of moist BC after 10 min was 44 times its own weight, thus higher than in the present study. The swelling increased gradually and reached 52 times its own weight after 12 h, while in our case, it reached only 1792%.

Both types of BC membranes swelled rapidly in the first half an hour. After this moment, BCm increased gradually in the next 6 h, then it maintained its swollen mass (Figure 4a). On the other hand, BCd increased gradually up to 24 h (Figure 4b). Similarly, Lin, Lien, Yeh, Yu, and Hsu [1] showed that moist BC membranes swelled gradually for 6 h, then maintained a stable state for the next 60 h. Unlike our study, Rambo, Recouvreux, Carminatti, Pitlovanciv, Antônio, and Porto [8] observed that oven-dried BC reached the maximum swelling ratio (175%) after 30 min, then decreased slightly to 153% between 0.5 and 1 h, and achieved a stable state after 1 h.

The swelling ratio over time of the BCd samples taken as a whole was significantly influenced by all three tested inoculum volumes, with the pellicle obtained with 5 mL achieving the highest swelling ratio (2121.73%). On the other hand, only the harvest days 12 and 18 significantly influenced the swelling ration of BCd samples taken as a whole. Six days of fermentation probably generated pellicles too thin with loose fibrillar network. In contrast, all BCm samples had comparable swelling ratios at every tested interval, thus no influence of the two fermentation variables was observed. However, when considering BCm as a batch, a significant influence of the swelling ratio over time by the inoculum volume was observed for the samples obtained with 5 mL (*p* < 0.0001). Similarly, only a fermentation period of 12 days seemed to significantly influence this relation.

The BCd batch, considered as a whole, showed significant differences in swelling rations up until 60 min after soaking, while BCm until 20 min (*p* < 0.001). Thus, BCm samples swelled significantly faster than BCd (bm = 22.07 vs. bd = 32.52), suggesting that the oven-drying processing affects the swelling ratio of BC by probably modifying the morphology of the pellicle network [1,7,17]. Thus, the oven-drying processing negatively affects the uptake speed of the BC membranes. Similar to our results, Sanchavanakit et al. [68] reported that BC membranes in a wet state had a swelling ratio up to 10 times higher (1027%) than air-dried BC (106%). Additionally, Alonso, Faria, Mohammadkazemi, Resnik, Ferreira, and Cordeiro [18] observed that the rehydration ability of freeze-dried BC is far superior (up to nine times) compared to oven-dried BC, which had a much lower swelling ratio capacity (50%). This could be due to pore preservation during manipulation, compared to oven-dried pellicles, which altered pore structure. The compact structure, due to drying, reduced the swelling ability. Thus, the processing techniques altered the BC pore availability and reduced the swelling ability of dried BC. In contrast, our results showed that BCd had the highest swelling ratio after 24 h (2121%) in the samples with 5 mL inoculum harvest on the 18th day, compared to the same BCm samples (1600%). This may be due to our reporting of the swelling ratios of both BCm and BCd to their initial weight and not to the initial dry mass.

Both tested fermentation variables, the inoculum volume and the harvest day, significantly influenced the swelling activity BC, as a whole, (*p* < 0.002). In general, a volume of inoculum of 1 mL produced pellicles with significant swelling up to a mean of 20 min, while higher volumes up to 30 min. This showed, once again, that the uptake ability of BC is influenced by the inoculum volume, most probably because of the difference in thickness of the pellicles (Supplementary Materials Table S2), their difference in network morphology, and fiber thickness. The thickness varied for all pellicles, according to the inoculum volume and harvest day, from 1.34 ± 0.20 mm on the 6th harvest day to 2.67 ± 0.67 mm on the 18th day (Supplementary Materials Table S2). Yoshino, Tabuchi, Uo, Tatsumi, Hideshima, Kondo, and Sekine [37] reported that BC expanded up to 80% in the first 10 min and about three times its initial thickness after 4 h, reaching a thickness of 2.5 mm. Juncu et al. [69] compared different films of sodium carboxymethyl cellulose with increasing BC content. The study revealed that the swelling degree decreased with the increase in BC content. A content of 12.5 mg of BC in the membranes showed the highest swelling ratio. In our case, the swelling ratio increased progressively and constantly during the 24 h, probably because our maximum cellulose content was 9.83 ± 1.46 mg, thus below the 12.5 inflection point.

BC mechanical properties were in indirect correlation with the swelling ratio. The maximum load, tensile strength (r = -0.534; *p* = 0.002), Young’s modulus, and stiffness (r = −0.506; r = 0.004) were negatively correlated with the swelling ability of the BC membranes. In contrast, the elongation at break is positively correlated to the uptake ability (r = 0.534; *p* = 0.002). A reduced water content in BC membranes brings the fibers closer together, which gives the tendency to assemble with each other into more compact fiber bundles leading to a more compact structure [7,17]. The uptaken water in BC pellicles limits the interactions among the fibers by competitive water–cellulose hydrogen bonds. Water in BC pellicle is crucial to achieving a good alignment of the BC fibers and, thus, good mechanical properties, since water can serve as a plasticizer [40].

The half-swelling time of BCd varied between 0.99 ± 0.38 and 2.12 ± 0.84 h, with significant differences among samples (Table 2). The fermentation had an impact, with samples harvested after 18 days having higher half-swelling times. For the BCm, this variation was between 2.47 ± 0.20 and 2.68 ± 0.18 h, but with no significant differences. Linear regression analysis showed that only significant influence on the half-swelling time of all BC samples seems to be the processing. Oven processing negatively affected the half-swelling time (b = −1.028), with a mean value of 1.5 h compared to 2.52 h for BCm.

An ideal wound dressing must have a high water uptake ability, to avoid the accumulation of wound exudate, which reduces the wound healing and permits the oxygenation to the wound [3,27]. Thus, the swelling ratio and half-swelling time are important parameters for BC intended as wound dressing. BC would need to absorb the exudate and maintain proper wound moisture during the healing process, which promotes the penetration of active substances embedded in the dressing and provides a painless removal after recovery [1]. Additionally, a high uptake ability is also needed for a dressing delivering controlled-release active substances [27,69]. These results are in accord with previous studies; Wei, Yang, and Hong [27] suggested that the highly porous structure permitted loading of drugs into the hydrogel fibrillar matrix. BC has shown to possess this ability, being, thus, suited for this biomedical use.

**Figure 5:**
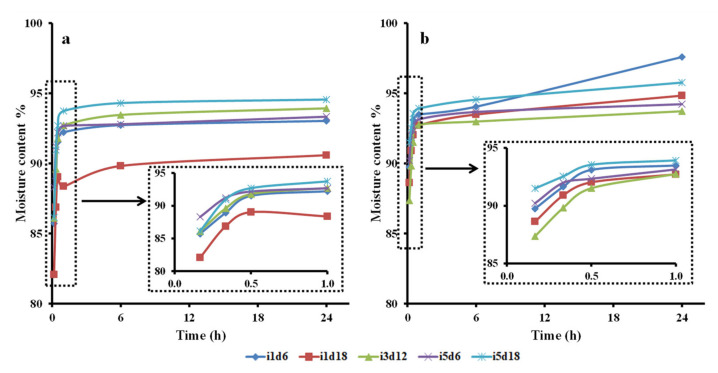
Moisture content of dry and moist BC membranes over 24 h. (**a**)—moist BC membrane; (**b**)—dry BC membrane; i—inoculum, d—harvest day; the number following “i” stands for inoculum volume 1, 3, 5 mL and the number following “d” represents the harvest day 6, 12, 18 days.

The moisture content of both BC membranes increased at the end compared with the beginning of the trials (Figure 5, Supplementary Materials Table S4). BCm and BCd moisture content was significantly different after 10 min and 24 h (*p* = 0.013; *p* = 0.033, respectively). The water content of BCd increased from 90% after 10 min to 95% in 24 h, compared to BCm, with a moisture content of 86% after 10 min and reaching 93% after 24 h. It was observed that the oven-drying processing significantly affected the moisture content of BC membranes (*p* = 0.012). BCm had a loosely interconnected network structure, compared to BCd as seen in Figures 8 and 9. Yet, the structure of oven-dried BC was more compact with no visible pores on the surface, as described by Ullah, Badshah, Mäkilä, Salonen, Shahbazi, Santos, and Khan [4], which explains the much lower moisture content of BCd compared to BCm. Lin, Lien, Yeh, Yu, and Hsu [1] showed similarly to our study that the moisture content of moist BC membranes increased gradually until 6 h, then they maintained a stable state for the next 60 h.

The diameter of the fibrils in the BC membranes is in direct correlation with the moisture content (r = 0.431; *p* = 0.018). The matrix of BC membranes consists of randomly arranged fibrils and a variety of empty space in between, which helps absorb and store water inside the membrane [1]. There was a positive correlation between BC moisture content and Young’s modulus and stiffness (r = 0.431 and r = 0.432; *p* = 0.017, respectively). Additionally, the moisture content is negatively correlated to the elongation at break (r = −0.454; *p* = 0.012). The results are consistent with other authors‘ reporting that the mechanical behavior of BC essentially depends on its moisture content [44].

BCd samples were significantly different after 10 min (*p* = 0.060). The pellicles obtained with 3 mL harvested after 12 days had the lowest moisture content (87%), while the membranes with 5 mL inoculum harvested after 18 days had the lowest moisture (91%) (Figure 5b). The moisture content over time of the BCd samples taken as a whole was significantly influenced by both tested variables, with the pellicle obtained with 1 mL harvested on the 6th day uptaking the highest moisture content of 96% (Figure 5b). Similarly, BCm samples taken as a whole were significantly influenced by all three tested inoculum volumes with the pellicles obtained with 5 mL (*p* < 0.0001) uptaking the highest moisture content (94%). On the other hand, only the harvest days 6 and 12 significantly influenced their moisture content. A fermentation period of 18 d probably generated pellicles too thick with a too dense fibrillar network (Figure 5a).

The half-moisture time of BCd and BCm membranes was significantly different (*p* = 0.010) (Supplementary Materials Table S6), and linear regression analysis showed that processing might be the only significant variables influencing the half-moisture time of all BC samples. Oven-drying processing affected the half-moisture time (b = 0.726) with a mean value of 1.15 h compared to 0.43 h for BCm. The half-moisture time of BCm membranes was in direct correlation with the half-swelling time (r = 0.844; *p* = <0.0001), because, logically, the more a pellicle swells, the higher its water content becomes.

#### 3.2.3. Drug Release

**Figure 6:**
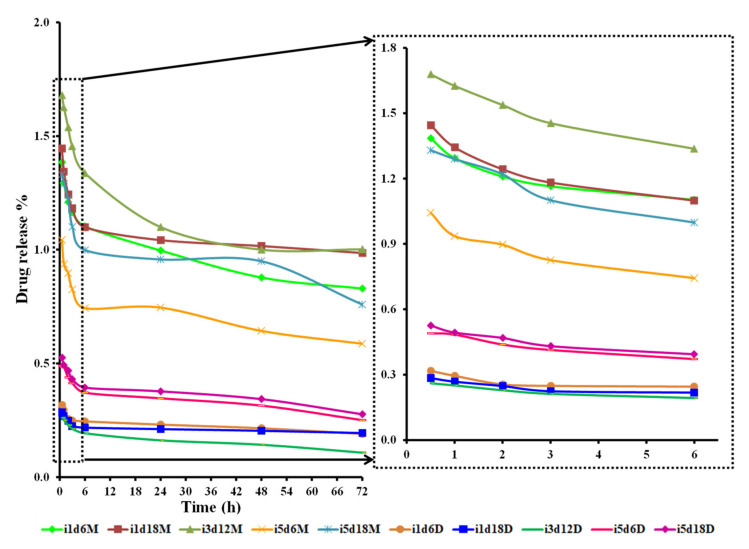
Drug release activity of BCd and BCm membranes over 72 h; i—inoculum, d—harvest day, M—moist membrane, D—dry membrane; the number following “i” stands for inoculum volume 1, 3, 5 mL and the number following “d” represents the harvest day 6, 12, 18 days.

The BCd and BCm membranes released significant amounts uptaken fluid (beet juice) during the 72 h (Figure 6, Supplementary Materials Table S5). Both BCd and BCm membranes showed a significant drug release capacity over time (*p* < 0.001). The release capacity was significantly influenced by all three inoculum volumes in both BCd (*p* < 0.005) and BCm (*p* < 0.046). The BCd samples obtained with 5 mL inoculum and harvested after 18 d had the highest drug release capacity (Figure 6), while BCm had the highest drug release obtained for pellicles obtained with 3 mL inoculum and fermented for 12 d (Figure 6). A gradual drug release was observed throughout the 72 h, with a significant difference between the two types of membranes throughout the tested period (*p* < 0.0001) (Figure 6). BCm samples had up to three times higher drug release capacity than BCd (bm = −0.006 vs. bd = -0.003), suggesting that the oven-drying processing negatively affected the drug release ability of BC. The membrane type (moist or dry) significantly influenced the release capacity of BC (*p* < 0.0001), thus BCm had an almost three times higher drug release capacity than BCd (Figure 6). It seems that oven-drying makes the structure of BC more rigid and less porous [7,17,18]. The three-dimensional porous structure of BCm can facilitate the drug release activity because of the larger surface area that facilitates drug uptake and has faster release rates [27]. Skvortsova, Gromovykh, Grachev, and Traskin [44] found that drying leads to distortions of the BC structure due to aggregation of cellulose fibrils. As a result, the porosity appears to decrease significantly. Contrary, Ullah, Badshah, Mäkilä, Salonen, Shahbazi, Santos, and Khan [4] reported almost similar drug release rates for both the freeze-dried and oven-dried BC with no effect of the drying method. Regardless of the processing method, almost 100% of the drug was released within 45 min. The immediate drug release is linked to the hydrophilic nature and swelling behavior of BC. Wei, Yang, and Hong [27] observed a stable and continuous release of the antimicrobial agent within 24 h.

Generally, the drug release from hydrogels can be influenced by many factors such as swelling, drug concentration, drug characteristics, and the hydrogel structure [2]. The highly porous structure of BCm (Figure 8) and BCd (Figure 9) can easily permit the loading of drugs into their matrix and subsequent drug release. It was observed that the samples obtained with 5 mL inoculum and harvested after 18 d had the highest swelling ratio in both BC membrane types, but BCm had a significantly better drug release behavior. BCd maximum drug release was obtained for the samples with 3 mL inoculum harvested on the 12th day. The rate of drug release depends on the water content of the swollen hydrogel and on the structure of the fibrillar network [27]. Juncu, Stoica-Guzun, Stroescu, Isopencu, and Jinga [69] stated that drug release decreased with the increase in BC content. Therefore, the concentration of BC could be an important factor, which may control drug release. 

Additionally, they observed that the release ability presented a sudden growth during the first 20 min. The dry BC films released about 66% of the drug within one day [27].

Both tested variables, the fermentation period and the inoculum volume, significantly influenced the drug release of both BC membranes. The inoculum volume significantly influenced the drug release capacity of both membranes up to 48 h. The release capacity over time of the BCm samples taken as a whole was significantly influenced by all three tested fermentation periods, compared to BCd samples, which were only influenced by the 6th and 12th days. BCd samples with 5 mL had significantly higher release ability. BCm release ability was also significantly influenced by 3 mL inoculum volume, which had the higher drug release, up to 6 h (Figure 6), with the highest values on the 12th harvest day compared to the 6th day.

The half-release time of BCd varied between 3.68 ± 0.32 and 12.78 ± 3.45 h, with significant differences among samples (Table 2). For the BCm, this variation was between 3.78 ± 1.76 and 8.60 ± 2.81 h, but with no significant differences. The inoculum volume affected the half-release time (b = 0.813, *p* = 0.008). An inoculum volume of 5 and 3 mL had a significant influence on half-release time of both membrane types (*p* = 0.002). The fermentation period only influenced the half-release time of BCd, with the highest value of 12.78 ± 3.45 h.

BC is an attractive material for the fabrication of intelligent adsorptive materials for drug-delivery applications because of its properties: an ultrafine fibrous network structure, a good water-absorbance capacity, and optimal mechanical properties [32]. BC appears to be a promising wound dressing material because of its ability to absorb exudate and controlled release of medical substances at the initial stage of wound healing [7,17,18].

#### 3.2.4. Mechanical Properties

Wound dressing materials need strength and flexibility and should maintain their integrity during use [1,3]. BCd with 1 mL inoculum harvested on the 18th day had the maximum load of 12.41 N and a tensile strength of 10.343 MPa, compared to the BCm samples, with a less than half that resistance of only 5.571 N and 4.642 MPa, respectively (Table 2, Supplementary Materials Table S6). The tensile strengths obtained in the current study for BCd is in agreement with previous studies where it ranged between 10.32 [30] and 48.17 ± 15.38 MPa [70]. Similarly, BCm tensile strengths of 14.77 [1] up to 207.0 MPa [24] were reported.

Among the BCd samples, only the 5 mL inoculum harvested on the 18th day membrane was significantly different, and only for Young’s modulus and the stiffness (*p* = 0.022). This sample had the highest values of 209.389 MPa and 41.865 kN/cm, respectively. In contrast, BCm samples significant higher values for maximum load and tensile strength (*p* = 0.007) were observed for the samples harvested on the 18th day.

The results of mechanical testing showed that there was a statistically significant difference between BCd and BCm, taken as two batches (*p* < 0.0001) (Table 2, Supplementary Materials Table S6). The maximum load and tensile strength were significantly higher in BCd membranes compared to BCm (*p* < 0.0001). The Young’s modulus and stiffness were significantly higher in BCd samples, compared to BCm (Table 2, Supplementary Materials Table S6). The BCd samples with 5 mL inoculum harvested on the 18th day had a Young’s modulus of 209.389 MPa, which was almost 10 times higher than the corresponding BCm samples. Our results are in line with other studies that reported a range from 131 [30] up to 458.38 MPa [70] for the Young’s modulus of BCd and 0.76 [3] to 33.57 MPa [1] for BCm.

BCm had significantly higher elongation at break values, up to twice higher than BCd (Supplementary Materials Table S6). The current report shows that the elongation at the break of BCd may vary between 2.26 ± 0.73% [71] and 9.00% [30], in contrast to BCm, which ranges between 4.54% [72] and can reach up to 32.17% [1]. This makes BCd membranes more brittle. This was in accord with our visual observation that BCm was more elastic and malleable, compared to BCd, which was inflexible and hard to bend. Similarly, Fu, Zhang, and Yang [7] stated that the dry BC film was more brittle than the moist BC film, and as a result, it broke easier in the tensile test. During elasticity testing, a decrease in strength occurs, leading to cracks in the BC membrane until it ruptures. Thus, the increase in crack propagation occurs very quickly in dry pellicles due to the lack of elasticity [70].

Nevertheless, the mechanical properties of BC may vary based on culturing and processing parameters, such as inoculum volume, culture time, medium, or post treatment [1]. Our results corroborate this statement, as the maximum load and tensile strength of the BCm was significantly influenced by the harvest day (*p* < 0.001). In contrast, the inoculum volume did not appear to influence the mechanical properties of BCm. In contrast, the properties of BCd did not seem to be influenced by the inoculum volume nor harvest day. It is observed, however, that the inoculum had a great influence on Young’s modulus and stiffness (*p* < 0.051). However, although close, it did not reach statistical significance.

#### 3.2.5. Scanning Electron Microscopy (SEM)

The surface morphology of the BCd and BCm membranes as seen by SEM is shown in Figures 8 and 9.

The structure is a well-organized, highly porous, three-dimensional fibrillar network, similar to previous reports [6,18,72]. The matrix of BC membranes consists of randomly arranged nanofibers and empty spaces distributed randomly in between. This network structure helps absorb and store water or water soluble compounds inside the membrane [1].

**Figure 7:**
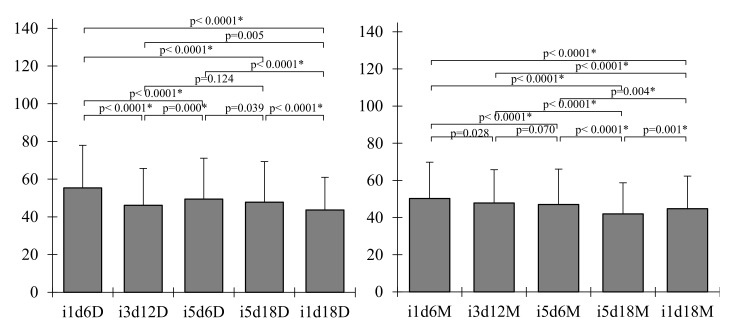
Kruskal–Wallis graphic test showing fiber diameter differences among BC dry and moist membranes. i—inoculum, d—harvest day, M—moist membrane, D—dry membrane; the number following “i” stands for inoculum volume 1, 3, 5 mL and the number following “d” represents the harvest day 6, 12, 18 days; the “*” indicates a statistical significance at the level indicated by *p*.

**Figure 8:**
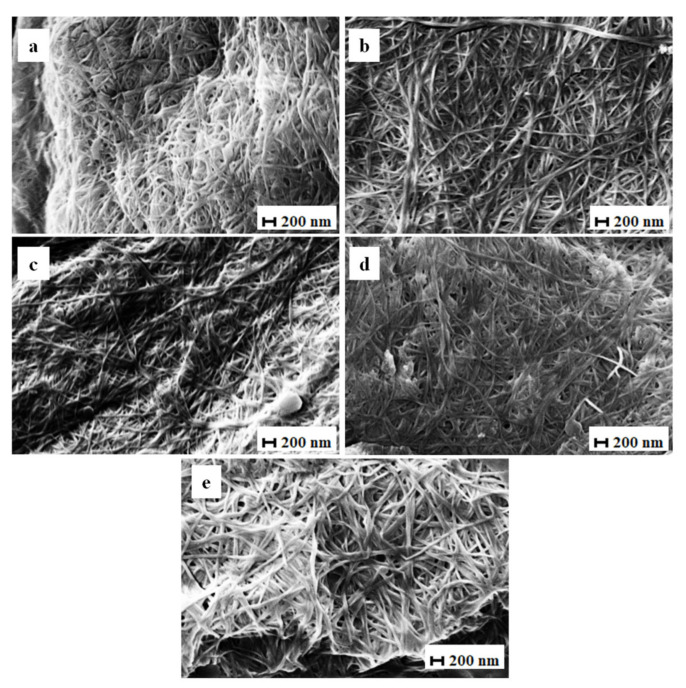
SEM images of moist BC. (**a**)—i1d6; (**b**)—i5d6; (**c**)—i1d18; (**d**)—i5d18; (**e**)—i3d12; i—inoculum, d—harvest day; the number following “i” stands for inoculum volume 1, 3, 5 mL and the number following “d” represents the harvest day 6, 12, 18 days (electron high tension (EHT) = 10.00 kV; magnitude (Mag) = 10.00 KX; working distance (WD) = 7.0 mm; Signal A = SE1).

The average fiber diameter of BCm ranged between 40.60 ± 4.99 and 49.30 ± 4.18 nm (Table 2, Figure 7), in accord with current research [30]. The largest median diameter was measured for membranes with 1 mL inoculum volume harvested on the 6th day (Figure 8a), significantly different from the membranes harvested on the 18th day with 5 mL of inoculum (Figure 8d). Both tested variables seemed to negatively influence the diameter of the BC fibrils, with the inoculum volume having a greater impact (b = −0.91; *p* = 0.013) than harvest day (b = −0.42; *p* = 0.001). Fu, Zhang, Li, Wu, Zhuo, Huang, Qiu, Zhou, and Yang [30] reported the fiber diameters of thick BC (138.6 ± 37.6 nm) and thin BC (189.2 ± 57.9 nm) and, similar to our results, showed that the diameter of the fibers decreased as the thickness of the membrane and the incubation period increased.

**Figure 9:**
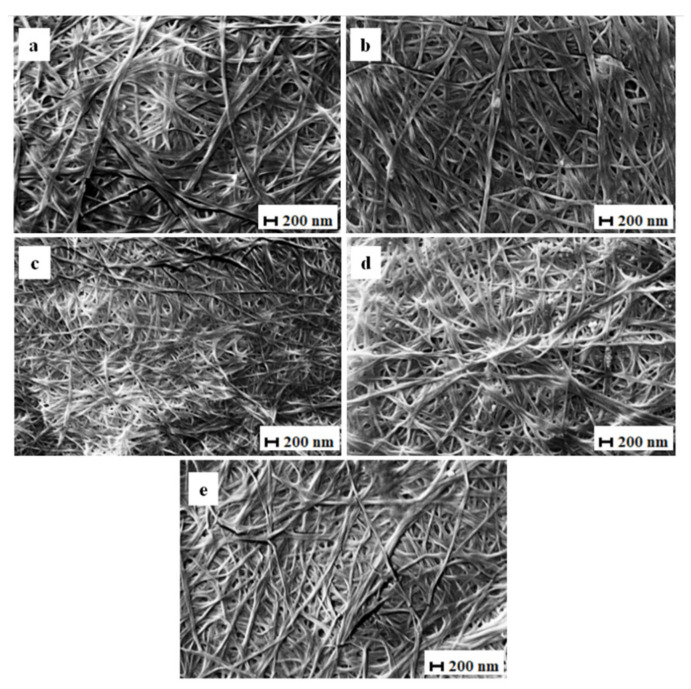
SEM images of dry BC. (**a**)—i1d6; (**b**)—i5d6; (**c**)—i1d18; (**d**)—i5d18; (**e**)—i3d12; i—inoculum, d—harvest day; the number following “i” stands for inoculum volume 1, 3, 5 mL and the number following “d” represents the harvest day 6, 12, 18 days. (electron high tension (EHT) = 10.00 kV; magnitude (Mag) = 10.00 KX; working distance (WD) = 7.0 mm; Signal A = SE1).

The fiber diameter of BCd ranged, on average, between 51.34 ± 6.99 and 41.40 ± 3.87 nm (Table 2), which is consistent with previous studies. Pourjavaher, Almasi, Meshkini, Pirsa, and Parandi [72] reported a fiber diameter around 45–70 nm, while Volova et al. [73] obtained a range of 52 to 173 nm for dried BC (freeze-dried and oven-dried). The median fiber diameter of BCd ranged between 41.40 nm in membranes with 1 mL inoculum volume harvested on the 18th day (Figure 9c) and 51.33 nm in membranes with the same inoculum volume but harvested on the 6th day (Figure 9a and Figure 7).

The latter diameter was significantly higher (*p* < 0.0001). For BCd, only the harvest day had a significant negative influence (b = −0.47; *p* = 0.014) similar in impact with BCm.

As reported by Zeng et al. [74], the drying method did not seem to influence the fibril diameter. This is surprising considering the differences in macroscopic structure between the two BC membranes types.

#### 3.2.6. Optimization of Bacterial Cellulose (BC) by Response Surface Methodology (RSM)

The properties of the two types of BC obtained with varying harvest periods and inoculum volumes as set by a BBD experimental design were analyzed by RSM, and the predicted values are shown in Table 2. The Mann–Whitney two-tailed test showed non-significant differences between the experimental and predicted values at a significance level of 0.001. The multiple regression analysis of the RSM generated a system of polynomial equations that proved to be good statistical models for the experimental attributes of BC (Table 3). The lack-of-fit of all obtained models showed was not significant at levels higher than *p* = 0.440 (Table 3). This suggests that the proposed models fit the data.

The model equations within the tested intervals (X_1_: 6…18 d and X_2_: 1…5 mL) in uncoded values for the membranes are presented in Table 4, Equations (4)–(15).

**Figure 10:**
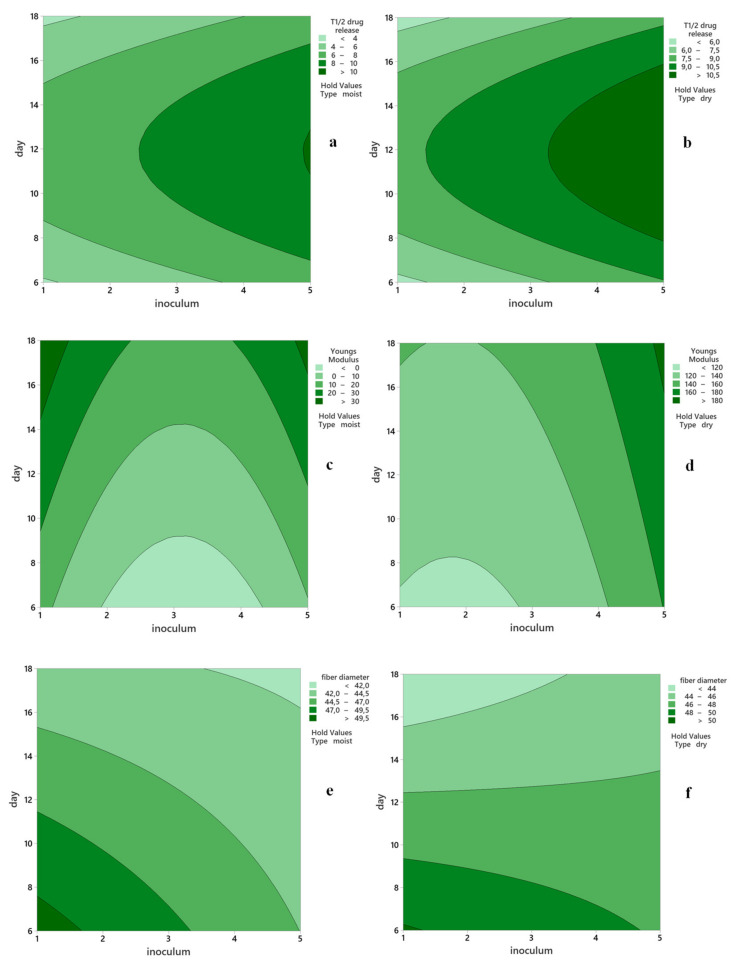
Response surface methodology RSM contour plots of drug half-release time of moist (**a**) and dried (**b**) bacterial cellulose (BC); Young’s modulus of moist (**c**) and dried (**d**) BC; fiber diameter of moist (**e**) and dried (**f**) BC.

The RSM contour plots show the relationship between the two continuous predictors (X_1_: harvest day and X_2_: inoculum volume) and the three fitted response variables (Y_3_: drug-release half-life; Y_5_: Young’s modulus; Y_6_: fiber diameter) for each of the two types of membranes (dry and moist) (Figure 10). By using the RSM, we generated a desirability function that enabled us to determine the mathematical optimum of the three parameters taken into consideration, within the proposed ranges.

The optimization procedure was run against the RSM model taken as a system of equations. The goal was to identify the values of the independent variables (X_1_ harvest day and X_2_ inoculum volume) that jointly optimize the fitted dependent variables set as follows: thickness—target to 2 mm; fiber diameter—minimum; swelling half-life—maximum; drug-release half-life—maximum; tensile strength—maximum; Young’s modulus—minimum. Thus, the system of equations has with only one possible solution, the optimal production conditions in terms of harvest day and inoculum volume. After computing the optimization analysis, our model suggested that the optimum conditions to obtain BC with appropriate properties for biomedical uses might be: X_1_: harvest day = 15.70, X_2_: inoculum volume = 5 mL, and X_3_: membrane type = moist, with a composite desirability = 0.60, when considering harvest intervals of 6 to 18 d, inoculum volumes of 1 to 5 mL, and moist or oven-dried types. Thus, based on the results obtained in the RSM optimization study the following optimum parameters were chosen for further model validation: 16 days of fermentation and 5 mL of inoculum (Table 5), which contains the predicted and the experimental values.

The optimization procedure was run again with X_3_: membrane type fixed on “dry” to obtain the optimal solution for BCd, as well. This resulted in X_1_: harvest day = 13.64, X_2_: inoculum volume = 3.60 mL, with a composite desirability = 0.58. Again, the values were approximated to for X_1_ = 14 d and X_2_ = 4 mL for ease of working. The predicted values for the BC properties were obtained after computing the model for the optimum conditions in each case.

As it can be observed in Table 5, all validation experimental results fited within the 95% PI intervals, with only one exception, the Young’s modulus of the oven-dried BC. Thus, the model for BCm was validated for all the five response variables, while the model for BCd was validated for four. By setting optimization goals in line with the requirements of a “wound dressing”-use scenario for BC, we obtained and validated the following values for the fermentation parameters: an inoculum volume of 5 mL and a harvest day of 16 for BCm, and an inoculum volume of 4 mL and a harvest day of 14 for BCd.

However, of the two types, we would recommend the moist type BC, not only because we managed to validate its production methods for all the five response characteristics, but also because of its superior mechanical properties, a unique fibrillar structure and water uptake and release properties, make it suitable for wound dressing [1,6,7].

## 4. Conclusions

The preliminary study showed that a combined 0.1 M NaOH and 3% NaOCl purification treatment was effective in whitening BC, and TEM imaging revealed that all bacterial cells were removed from BC pellicle matrix. Additionally, the inoculum volume and the harvest day were shown to significantly influence the thickness of the membranes (*p* < 0.008 and *p* < 0.0001, correspondingly). An increase in the fermentation period significantly increased BC dry weight (*p* < 0.002), water content (*p* < 0.0001), and yield (*p* = 0.002), while the uniformity was not influenced by the inoculum or the fermentation period.

For the optimization study, a Box–Behnken design was used to assess the effect of inoculum volume and fermentation on two BC processing methods. By setting the optimization goals in line with the requirements of a “wound dressing”-use scenario for BC, we obtained and validated the following values for the fermentation parameters: an inoculum volume of 5 mL and a harvest day of 16 for BCm, and an inoculum volume of 4 mL and a harvest day of 14 for BCd. We managed to validate the production method for all the five response characteristics of BCm. For a “wound dressing”-use scenario of the two types, we would recommend the moist type BCm because of its fibrillar network that was able to incorporate bioactive compounds (half-swelling time of 1.930 h) and optimally release them (drug half-release time of 3.678 h). This would lead to a hastening healing of wounds. Additionally, the obtained BCm has good tension strength (4.770 MPa) and low elongation at break (13.305 MPa), which would make the BC an easy to work with material.

## Figures and Tables

**Table 1:** Coded coefficients of the response surface regression of thickness (Y_1_) performed on the factorial design with 5 levels and regression equation in uncoded units.

Model/Term	Linear	b0	b1	b2	Square	b11	b22	Lack-of-Fit	R^2^
Coefficient		1.966	0.393	−0.047		−0.130	0.167	0.431	0.50
*p*-value	0.000	0.000	0.000	0.354	0.054	0.143	0.053		
Regression equation:	Y_1_ = 1.123 + 0.149 · X_1_ − 0.274 · X_2_ − 0.004 · X_1_^2^ + 0.042 · X_2_^2^								

Note: The explanatory variables were X_1_: harvest (d) and X_2_: inoculum volume (mL). A stepwise selection of terms was used with α ≤ 0.15 for a hierarchical model.

**Table 2:** The properties of bacterial cellulose (BC) of interest for biomedical purposes at different fermentation conditions based on a Box–Behnken design for response surface methodology (RSM).

	Independent Variables			Response—Dependent Variables												Desir
	X_1_ Harvest (d)	X_2_ Inoculum Volume (mL)	X_3_ BC Type	Y_1_ Thickness * (mm)		Y_2_ Half-Swelling Time (h)		Y_3_ Drug Half-Release Time (h)		Y_4_ Tensile Strength σ (MPa)		Y_5_ Young’s Modulus E (MPa)		Y_6_ Fiber Diameter (nm)		
				exp	pred **	exp	pred **	exp	pred **	exp	pred **	exp	pred **	exp	pred **	
1	6	1	dry	1.68 ± 0.16 ^bc^	1.62	1.25 ± 0.5 ^bc^	1.11	4.95 ± 0.77 ^de^	5.63	7.61 ± 0.21 ^ab^	7.88	128.92 ± 30.37 ^b^	118.19	51.34 ± 6.99 ^a^	50.18	0.40
2	18	1	dry	2.67 ± 0.67 ^ab^	2.81	1.92 ± 0.89 ^ab^	1.89	3.68 ± 0.32 ^e^	5.36	10.34 ± 3.69 ^a^	9.85	139.34 ± 22.35 ^b^	142.08	41.40 ± 3.87 ^de^	42.40	0.48
3	12	3	dry	2.09 ± 0.15 ^ab^	1.93	0.99 ± 0.38 ^c^	1.50	12.78 ± 3.45 ^a^	10.28	10.04 ± 1.90 ^a^	8.86	117.86 ± 28.18 ^b^	133.85	46.00 ± 7.61 ^bcd^	46.33	0.57
4	6	5	dry	1.34 ± 0.15 ^c^	1.05	1.22 ± 0.6 ^bc^	1.11	9.12 ± 1.60 ^b^	8.88	7.08 ± 2.78 ^abc^	7.88	143.99 ± 36.54 ^a^	160.75	47.11 ± 8.77 ^abc^	47.82	0.41
5	18	5	dry	2.28 ± 0.23 ^ab^	2.23	2.12 ± 0.84 ^a^	1.89	8.25 ± 1.61 ^bcd^	8.61	9.22 ± 3.33 ^a^	9.85	209.39 ± 23.85 ^c^	184.64	45.78 ± 6.05 ^bcd^	44.90	0.53
6	6	1	moist	1.68 ± 0.16 ^bc^	1.62	2.47 ± 0.20 ^a^	2.52	5.93 ± 0.58 ^bcde^	3.81	3.02 ± 0.64 ^d^	2.45	16.03 ± 2.97 ^c^	13.26	49.30 ± 4.18 ^ab^	50.54	0.29
7	18	1	moist	2.67 ± 0.67 ^ab^	2.81	2.53 ± 0.28 ^a^	2.54	3.77 ± 1.76 ^e^	3.54	4.64 ± 0.32 ^bcd^	4.42	26.38 ± 15.22 ^c^	37.15	43.67 ± 4.19 ^cde^	42.75	0.41
8	12	3	moist	2.09 ± 0.15 ^ab^	1.93	2.68 ± 0.18 ^a^	2.53	5.97 ± 2.25 ^bcde^	8.46	2.91 ± 0.83 ^d^	3.44	21.59 ± 11.90 ^c^	5.60	45.49 ± 2.64 ^bcde^	44.83	0.54
9	6	5	moist	1.34 ± 0.15 ^c^	1.05	2.49 ± 0.38 ^a^	2.52	5.38 ± 1.95 ^cde^	7.06	2.61 ± 0.38 ^d^	2.45	12.44 ± 0.73 ^c^	9.18	45.12 ± 6.03 ^bcde^	44.48	0.36
10	18	5	moist	2.28 ± 0.23 ^ab^	2.23	2.47 ± 0.08 ^a^	2.54	8.60 ± 2.81 ^bc^	6.79	4.00 ± 0.55 ^cd^	4.42	21.82 ± 2.47 ^c^	33.07	40.60 ± 4.99 ^e^	41.56	0.57
*p*-value ***				0.992		0.824		0.853		0.897		0.971		0.912		

Where exp—experimental values; pred—values predicted by the RSM model; desir—overall desirability (0…1); *—the thickness was measured for the entire batch, before drying (n = 6); ** the predicted value resulted from the model optimizing the BC properties; *** Mann–Whitney two-tailed test (α = 0.001) of the experimental data versus the values predicted by the model optimizing the BC properties. Note: The data are presented as mean ± SD. Different letters (a–e within the same column show significant differences among the samples (Fisher (LSD), *p* < 0.05).

**Table 3:** Model parameters (coded coefficients), *p* values, and goodness-of-fit statistics obtained by response surface methodology (RSM) for each of the 6 response variables (Yi).

		Y_1_ Thickness (mm)		Y_2_ Half-Swelling Time (h)		Y_3_ Drug Half-Release Time (h)		Y_4_ Tensile Strength σ (MPa)		Y_5_ Young’s Modulus E (MPa)		Y_6_ Fiber Diameter (nm)		Desirability	
		coef	*p*	coef	*p*	coef	*p*	coef	*p*	coef	*p*	coef	*p*	coef	*p*
intercept	b0	1.926 ***	0.000	2.013 ***	0.000	9.370 ***	0.000	6.149 ***	0.000	69.720 ***	0.000	45.580 ***	0.000	0.553 ***	0.000
linear	b1	0.591 ***	0.002	0.203 *	0.054	−0.135	0.793	0.985 **	0.012	11.940 **	0.025	−2.677 ***	0.000	0.066 ***	0.000
	b2	−0.289 ***	0.000	NA	NA	1.627^***^	0.004	NA	NA	9.620 *	0.066	−0.889 *	0.132	0.0339 ***	0.000
	b3 (dry)	NA	NA	−0.514 ***	0.000	0.911 *	0.056	2.711 ***	0.000	64.120 ***	0.000	0.747	0.156	0.0216 ***	0.000
interaction	b12	NA	NA	NA	NA	NA	NA	NA	NA	NA	NA	1.215 **	0.044	0.0166 ***	0.000
	b13	NA	NA	0.191 *	0.068	NA	NA	NA	NA	NA	NA	NA	NA	−0.017 ***	0.000
	b23	NA	NA	NA	NA	NA	NA	NA	NA	11.660 **	0.028	0.924 *	0.118	−0.0219 ***	0.000
square	b11	NA	NA	NA	NA	−3.16 *	0.010	NA	NA	NA	NA	NA	NA	NA	NA
	b22	NA	NA	NA	NA	NA	NA	NA	NA	17.600 *	0.129	NA	NA	−0.123 ***	0.000
	b33	NA	NA	NA	NA	NA	NA	NA	NA	NA	NA	NA	NA	NA	NA
R^2^		0.70	-	0.61	-	0.47	-	0.73	-	0.90	-	0.59	-	0.995	-
Lack-of-fit		-	0.954	-	0.638	-	0.455	-	0.886	-	0.440	-	0.590	-	-
The model		-	0.000	-	0.000	-	0.003	-	0.000	-	0.000	-	0.000	-	0.000

Note: The explanatory variables were coef—coded coefficients, X_1_: harvest (d), X_2_: inoculum volume (mL), X_3_: membrane type. A stepwise selection of terms was used with α ≤ 0.15 for a hierarchical model; NA—not applicable, the parameter was removed from the model. * significant at *p* < 0.15, ** significant at *p* < 0.05, *** significant at *p* < 0.01.

**Table 4:** The model equations in uncoded values of response variables for each of the 2 types of membranes (dry and moist).

**Dry Bacterial Cellulose:**	
Y_2_ = 0.711 + 0.066·X_1_	(4)
Y_3_ = −4.540 + 2.086·X_1_+ 0.813·X_2_ − 0.088·X_1_^2^	(5)
Y_4_ = 6.891 + 0.164·X_1_	(6)
Y_5_ = 117.600 − 15.700·X_2_ + 1.991·X_1_ + 4.390·X_2_^2^	(7)
Y_6_ = 55.270 − 0.750·X_1_ − 1.197·X_2_ + 0.101·X_1_·X_2_	(8)
D = 0.232 + 0.173·X_2_ + 0.004·X_1_ - 0.031·X_2_^2^ + 0.001·X_2_*X_1_	(9)
**Moist bacterial cellulose:**	
Y_2_ = 2.504 + 0.002·X_1_	(10)
Y_3_ = −6.36 + 2.086·X_1_ + 0.813·X_2_ − 0.0879·X_1_^2^	(11)
Y_4_ = 1.468 + 0.164·X_1_	(12)
Y_5_ = 24.300 + 1.991·X_1_ − 27.400·X_2_ + 4.390·X_2_^2^	(13)
Y_6_ = 56.550 − 0.750·X_1_ − 2.121·X_2_ + 0.101·X_1_·X_2_	(14)
D = 0.055 + 0.010·X_1_ + 0.195·X_2_ + 0.001·X_2_·X_1_ − 0.031·X_2_^2^	(15)

Where X_1_—harvest (d); X_2_—inoculum volume (mL); Y_2_—half-swelling time (h); Y_3_—drug half-release time (h); Y_4_—tensile strength, (MPa); Y_5_—Young’s modulus (MPa); Y_6_—fiber diameter (nm); D—desirability function.

**Table 5:** Experimental validation of the mathematical model.

X_1_ = 16 d, X_2_ = 5 mL, and X_3_ = moist					X_1_ = 14 d, X_2_ = 4 mL, and X_3_ = dry			
Resp.	pred.	95% CI	95% PI	exp.	pred.	95% CI	95% PI	exp.
Y_1_	2.002	(1.751; 2.252)	(1.134; 2.869)	2.110	2.001	(1.835; 2.168)	(1.154; 2.848)	1.960
Y_2_	2.535	(2.217; 2.852)	(1.474; 3.595)	1.930	1.607	(1.334; 1.880)	(0.559; 2.654)	0.900
Y_3_	8.800	(6.740; 10.870)	(3.270; 14.34)	3.678	10.500	(8.300; 12.700)	(4.91; 16.08)	7.144
Y_4_	4.045	(2.992; 5.098)	(0.230; 7.860)	4.770	9.129	(8.161; 10.097)	(5.337; 12.921)	8.126
Y_5_	28.500	(7.400; 49.600)	(6.300; 83.200)	13.305	145.000	(123.400; 166.600)	(90.100; 200.000)	62.776
Y_6_	42.120	(39.660; 44.580)	(35.850; 48.390)	43.20	45.705	(44.101; 47.310)	(39.721; 51.689)	45.880

Where resp.—response variable; pred.—value predicted by the model; exp.—value resulted from the validation experiment; Y_1_—thickness (mm); Y_2_—half-swelling time (h); Y_3_—drug half-release time (h); Y_4_—tensile strength, (MPa); Y_5_—Young’s modulus (MPa); Y_6_—fiber diameter (nm).

## Supplementary Materials

The following are available online at https://www.mdpi.com/2073-4360/13/13/2088/s1, Figure S1 and Table S1: Principal component analysis of the effect of harvest day and inoculum volume on film thickness, uniformity, weight, and yield; Table S2: Characteristics of the bacterial cellulose pellicle dependent on the incubation and inoculum volume (thickness, uniformity, dry weight, water content, and yield); Table S3: Swelling ratio over time of the bacterial cellulose membranes dependent on the incubation and inoculum volume; Table S4: Moisture content over time of the bacterial cellulose membranes dependent on the incubation period and inoculum volume; Table S5. Drug release over time of the bacterial cellulose membranes dependent on the incubation period and inoculum volume; Table S6: Characteristics of dry and moist bacterial cellulose membranes dependent on the harvest day and inoculum volume (half moisture time, maximum load, elongation at break, and stiffness).
