# Correction: Bodea et al. Optimization of Moist and Oven-Dried Bacterial Cellulose Production for Functional Properties. *Polymers* 2021, *13*, 2088

**DOI:** 10.3390/polym13213821

**Published:** 2021-11-05

**Authors:** Ioana M. Bodea, Florin I. Beteg, Carmen R. Pop, Adriana P. David, Mircea Cristian Dudescu, Cristian Vilău, Andreea Stănilă, Ancuța M. Rotar, Giorgiana M. Cătunescu

**Affiliations:** 1 Department of Preclinical and Clinical Sciences, Faculty of Veterinary Medicine, University of Agricultural Sciences and Veterinary Medicine Cluj-Napoca, 400372 Cluj-Napoca, Romania; ioana.bodea@usamvcluj.ro (I.M.B.); florin.beteg@usamvcluj.ro (F.I.B.); 2 Department of Food Science, Faculty of Food Science and Technology, University of Agricultural Sciences and Veterinary Medicine Cluj-Napoca, 400372 Cluj-Napoca, Romania; andreea.stanila@usamvcluj.ro (A.S.); anca.rotar@usamvcluj.ro (A.M.R.); 3 Department of Technical and Soil Sciences, Faculty of Agriculture, University of Agricultural Science and Veterinary Medicine Cluj-Napoca, 400372 Cluj-Napoca, Romania; adriana.david@usamvcluj.ro; 4 Department of Mechanical Engineering, Technical University of Cluj-Napoca, 400114 Cluj-Napoca, Romania; mircea.dudescu@rezi.utcluj.ro (M.C.D.); cristian.vilau@tcm.utcluj.ro (C.V.)

The authors wish to make a change to the published paper [1]. In Table 2 and Table 3, the units of Fiber Diameter have been changed from “µm” to “nm”. Also, in the footnotes of Tables 4 and 5, the units of Fiber Diameter have been changed from “µm” to “nm”. The mistake was due to the authors’ oversight. To avoid misleading readers, we would like to update the data in the article. The authors apologize for any inconvenience this may have caused.

The manuscript will be updated, and the original will remain online on the article webpage: https://www.mdpi.com/2073-4360/13/13/2088.

## Figures and Tables

**Table 2:** The properties of bacterial cellulose (BC) of interest for biomedical purposes at different fermentation conditions based on a Box–Behnken design for response surface methodology (RSM).

	Independent Variables			Response—Dependent Variables												Desir
	X_1_ Harvest (d)	X_2_ Inoculum Volume (mL)	X_3_ BC Type	Y_1_ Thickness * (mm)		Y_2_ Half-Swelling Time (h)		Y_3_ Drug Half-Release Time (h)		Y_4_ Tensile Strength σ (MPa)		Y_5_ Young’s Modulus E (MPa)		Y_6_ Fiber Diameter (nm)		
				exp	pred **	exp	pred **	exp	pred **	exp	pred **	exp	pred **	exp	pred **	
1	6	1	dry	1.68 ± 0.16 ^bc^	1.62	1.25 ± 0.5 ^bc^	1.11	4.95 ± 0.77 ^de^	5.63	7.61 ± 0.21 ^ab^	7.88	128.92 ± 30.37 ^b^	118.19	51.34 ± 6.99 ^a^	50.18	0.40
2	18	1	dry	2.67 ± 0.67 ^ab^	2.81	1.92 ± 0.89 ^ab^	1.89	3.68 ± 0.32 ^e^	5.36	10.34 ± 3.69 ^a^	9.85	139.34 ± 22.35 ^b^	142.08	41.40 ± 3.87 ^de^	42.40	0.48
3	12	3	dry	2.09 ± 0.15 ^ab^	1.93	0.99 ± 0.38 ^c^	1.50	12.78 ± 3.45 ^a^	10.28	10.04 ± 1.90 ^a^	8.86	117.86 ± 28.18 ^b^	133.85	46.00 ± 7.61 ^bcd^	46.33	0.57
4	6	5	dry	1.34 ± 0.15 ^c^	1.05	1.22 ± 0.6 ^bc^	1.11	9.12 ± 1.60 ^b^	8.88	7.08 ± 2.78 ^abc^	7.88	143.99 ± 36.54 ^a^	160.75	47.11 ± 8.77 ^abc^	47.82	0.41
5	18	5	dry	2.28 ± 0.23 ^ab^	2.23	2.12 ± 0.84 ^a^	1.89	8.25 ± 1.61 ^bcd^	8.61	9.22 ± 3.33 ^a^	9.85	209.39 ± 23.85 ^c^	184.64	45.78 ± 6.05 ^bcd^	44.90	0.53
6	6	1	moist	1.68 ± 0.16 ^bc^	1.62	2.47 ± 0.20 ^a^	2.52	5.93 ± 0.58 ^bcde^	3.81	3.02 ± 0.64 ^d^	2.45	16.03 ± 2.97 ^c^	13.26	49.30 ± 4.18 ^ab^	50.54	0.29
7	18	1	moist	2.67 ± 0.67 ^ab^	2.81	2.53 ± 0.28 ^a^	2.54	3.77 ± 1.76 ^e^	3.54	4.64 ± 0.32 ^bcd^	4.42	26.38 ± 15.22 ^c^	37.15	43.67 ± 4.19 ^cde^	42.75	0.41
8	12	3	moist	2.09 ± 0.15 ^ab^	1.93	2.68 ± 0.18 ^a^	2.53	5.97 ± 2.25 ^bcde^	8.46	2.91 ± 0.83 ^d^	3.44	21.59 ± 11.90 ^c^	5.60	45.49 ± 2.64 ^bcde^	44.83	0.54
9	6	5	moist	1.34 ± 0.15 ^c^	1.05	2.49 ± 0.38 ^a^	2.52	5.38 ± 1.95 ^cde^	7.06	2.61 ± 0.38 ^d^	2.45	12.44 ± 0.73 ^c^	9.18	45.12 ± 6.03 ^bcde^	44.48	0.36
10	18	5	moist	2.28 ± 0.23 ^ab^	2.23	2.47 ± 0.08 ^a^	2.54	8.60 ± 2.81 ^bc^	6.79	4.00 ± 0.55 ^cd^	4.42	21.82 ± 2.47 ^c^	33.07	40.60 ± 4.99 ^e^	41.56	0.57
*p*-value ***				0.992		0.824		0.853		0.897		0.971		0.912		

Where exp—experimental values; pred—values predicted by the RSM model; desir—overall desirability (0…1); *—the thickness was measured for the entire batch, before drying (n = 6); ** the predicted value resulted from the model optimizing the BC properties; *** Mann–Whitney two-tailed test (α = 0.001) of the experimental data versus the values predicted by the model optimizing the BC properties. Note: The data are presented as mean ± SD. Different letters (a–e within the same column show significant differences among the samples (Fisher (LSD), *p* < 0.05).

**Table 3:** Model parameters (coded coefficients), *p* values, and goodness-of-fit statistics obtained by response surface methodology (RSM) for each of the 6 response variables (Yi).

		Y_1_ Thickness (mm)		Y_2_ Half-Swelling Time (h)		Y_3_ Drug Half-Release Time (h)		Y_4_ Tensile Strength σ (MPa)		Y_5_ Young’s Modulus E (MPa)		Y_6_ Fiber Diameter (nm)		Desirability	
		coef	*p*	coef	*p*	coef	*p*	coef	*p*	coef	*p*	coef	*p*	coef	*p*
intercept	b0	1.926 ***	0.000	2.013 ***	0.000	9.370 ***	0.000	6.149 ***	0.000	69.720 ***	0.000	45.580 ***	0.000	0.553 ***	0.000
linear	b1	0.591 ***	0.002	0.203 *	0.054	−0.135	0.793	0.985 **	0.012	11.940 **	0.025	−2.677 ***	0.000	0.066 ***	0.000
	b2	−0.289 ***	0.000	NA	NA	1.627 ***	0.004	NA	NA	9.620 *	0.066	−0.889 *	0.132	0.0339 ***	0.000
	b3 (dry)	NA	NA	−0.514 ***	0.000	0.911 *	0.056	2.711 ***	0.000	64.120 ***	0.000	0.747	0.156	0.0216 ***	0.000
interaction	b12	NA	NA	NA	NA	NA	NA	NA	NA	NA	NA	1.215 **	0.044	0.0166 ***	0.000
	b13	NA	NA	0.191 *	0.068	NA	NA	NA	NA	NA	NA	NA	NA	−0.017 ***	0.000
	b23	NA	NA	NA	NA	NA	NA	NA	NA	11.660 **	0.028	0.924 *	0.118	−0.0219 ***	0.000
square	b11	NA	NA	NA	NA	−3.16 *	0.010	NA	NA	NA	NA	NA	NA	NA	NA
	b22	NA	NA	NA	NA	NA	NA	NA	NA	17.600 *	0.129	NA	NA	−0.123 ***	0.000
	b33	NA	NA	NA	NA	NA	NA	NA	NA	NA	NA	NA	NA	NA	NA
R^2^		0.70	-	0.61	-	0.47	-	0.73	-	0.90	-	0.59	-	0.995	-
Lack-of-fit		-	0.954	-	0.638	-	0.455	-	0.886	-	0.440	-	0.590	-	-
The model		-	0.000	-	0.000	-	0.003	-	0.000	-	0.000	-	0.000	-	0.000

Note: The explanatory variables were coef—coded coefficients, X_1_: harvest (d), X_2_: inoculum volume (mL), X_3_: membrane type. A stepwise selection of terms was used with α ≤ 0.15 for a hierarchical model; NA—not applicable, the parameter was removed from the model. * significant at *p* < 0.15, ** significant at *p* < 0.05, *** significant at *p* < 0.01.
