# Supplementary material for: Optimization of Moist and Oven-Dried Bacterial Cellulose Production for Functional Properties
Source: Polymers (Basel). 2021 Jun 24;13(13):2088. doi: 10.3390/polym13132088 (PMC8272063; doi:10.3390/polym13132088)
Supplement: Supplementary file 1 [file polymers-13-02088-s001.zip › polymers-1250705-supplementary.pdf]

Optimization of Moist and Oven-Dried Bacterial Cellulose Production for Functional Properties

Ioana M. Bodea [1], Florin I. Beteg [1], Carmen R. Pop [2,*], Adriana P. David [3], Mircea Cristian Dudescu [4], Cristian Vilău [4], Andreea Stănilă [2], Ancuța M. Rotar [2] and Giorgiana M. Cătunescu [3,*]

[1] Department of Preclinical and Clinical Sciences, Faculty of Veterinary Medicine, University of Agricultural Sciences and Veterinary Medicine Cluj-Napoca, 400372 Cluj-Napoca, Romania;
ioana.bodea@usamvcluj.ro (I.M.B.); florin.beteg@usamvcluj.ro (F.I.B.)
[2] Department of Food Science, Faculty of Food Science and Technology, University of Agricultural Sciences and Veterinary Medicine Cluj-Napoca, 400372 Cluj-Napoca, Romania; andreea.stanila@usamvcluj.ro (A.S.); anca.rotar@usamvcluj.ro (A.M.R.)
[3] Department of Technical and Soil Sciences, Faculty of Agriculture, University of Agricultural Science and Veterinary Medicine Cluj-Napoca, 400372 Cluj-Napoca, Romania; adriana.david@usamvcluj.ro
[4] Department of Mechanical Engineering, Technical University of Cluj-Napoca, 400114 Cluj-Napoca, Romania; mircea.dudescu@rezi.utcluj.ro (M.C.D.); cristian.vilau@tcm.utcluj.ro (C.V.)
* Correspondence: carmen-rodica.pop@usamvcluj.ro (C.R.P.); giorgiana.catunescu@usamvcluj.ro (G.M.C.)

PRELIMINARY STUDY

Principal component analysis of the effect of harvest day and inoculum volume on film thickness, uniformity, weight, and yield

The principal component analysis (PCA) biplots (Figure 3) shows the distribution of samples and the influence of inoculum volume and harvest period on BC properties (thickness, uniformity, dry weight, water content and yield). The first 3 principal factors were selected: F1 consisting of dry weigh, water content, and thickness and accounted for 53.48% of the variability in the samples; F2 consisting of yield with 22.15% of the variability; and F3 consisting of uniformity with 14.45% of the variability (Table 3).

This grouping showed the positive correlation among dry weigh, water content, and thickness ($r > 0.53$; $p < 0.006$), and among water content, thickness, and uniformity ($r > 0.42$; $p < 0.037$). The samples are grouped mainly by the harvest day on both biplots, showing once again the strong influence of this parameter upon the selected properties of BC. Additionally, the confidence ellipses around each group of samples shows the significant differences among samples harvested at the beginning of the study period compared to those harvested after 15 days.

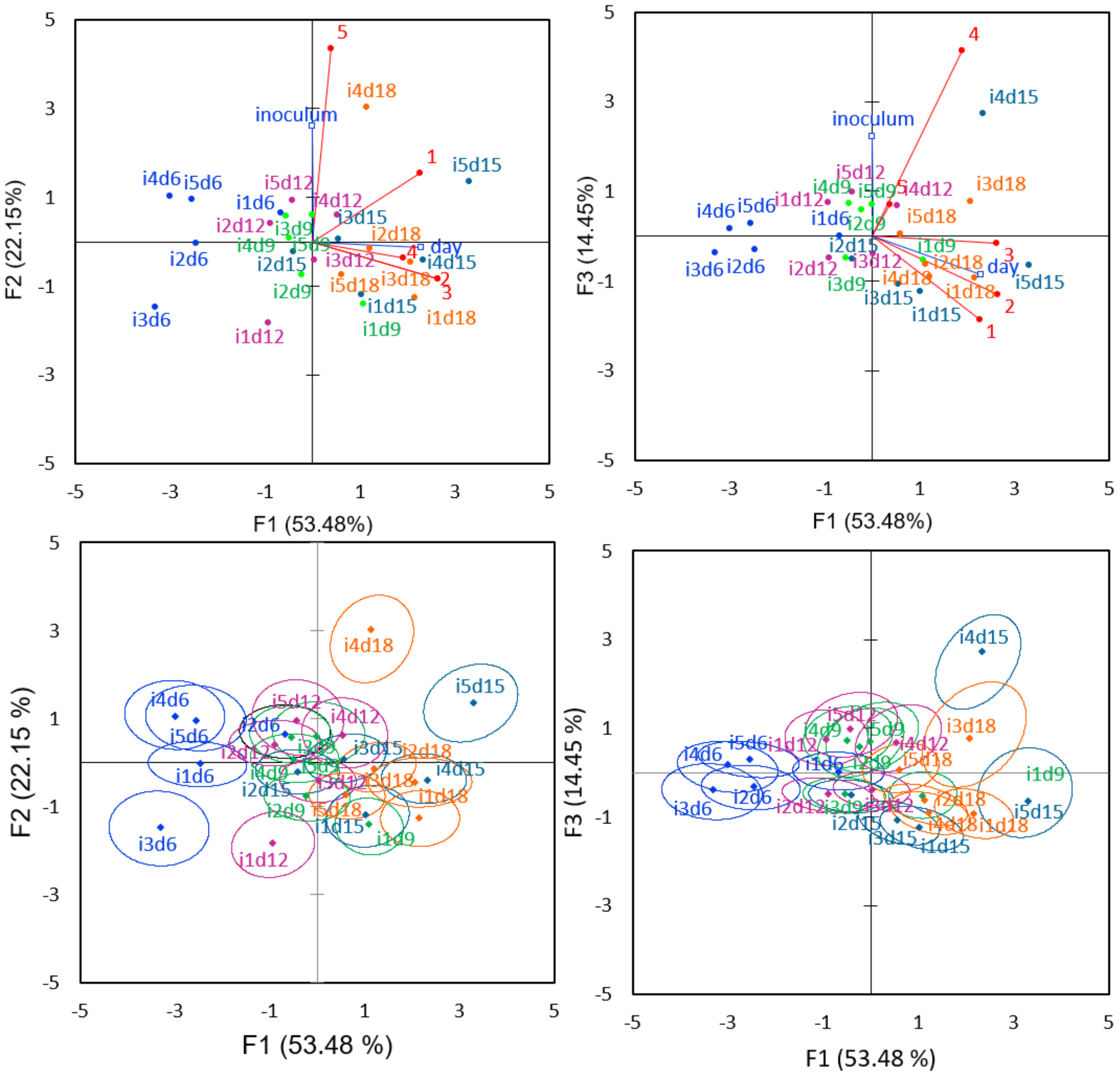

Figure S1. Principal component analysis (PCA) biplots of the samples and analyzed parameters of bacterial cellulose (axes F1 and F2: 75.63%; F1 and F3: 67.93%) where: 1 - dry weigh; 2 - water content; 3 - thickness; 4 - uniformity; 5 - yield; i – inoculum, d – harvest day; the number following “i” stands for inoculum volume 1, 3, 5 mL and the number following “d” represents the harvest day 6, 12, 18 days.

Table S1. Correlations between variables and Principal component analysis (PCA) factors, contribution of the variables and squared cosines of the variables.

PCA variable	Correlations between variables and PCA factors			Contribution of the variables (%)			Correlations between variables and PCA factors		
	F1	F2	F3	F1	F2	F3	F1	F2	F3
dry weigh	0.77	0.34	-0.33	22.39	10.33	15.24	**0.60**	0.11	0.11
water content	0.91	-0.19	-0.23	30.75	3.10	7.51	**0.82**	0.03	0.05
thickness	0.90	-0.19	-0.03	30.42	3.10	0.12	**0.81**	0.03	0.00
uniformity	0.65	-0.08	0.74	15.82	0.55	74.93	0.42	0.01	**0.54**
yield	0.13	0.96	0.13	0.63	82.92	2.20	0.02	**0.92**	0.02
inoculum	0.00	0.58	0.40				0.00	**0.33**	0.16
day	0.78	-0.03	-0.15				**0.62**	0.00	0.02

Note: Values in bold correspond for each variable to the factor for which the squared cosine is the largest.

Table S2. Characteristics of the bacterial cellulose pellicle dependent on the incubation and inoculum volume, mean±SD.

Harvest day (d)	Inoculum volume (mL)	Thickness (mm)	Uniformity (mm)	Dry weigh (mg)	Water Content (mg)	Yield (g/L)
	1	1.68±0.16^{fgh}	0.281±0.165^{bcd}	5.80±3.83^{bcd}	790.73±58.36^{bcdefg}	0.23±0.15^{bcd}
	2	1.41±0.32^{h}	0.189±0.115^{cd}	4.03±2.94^{bcde}	702.07±139.44^{defg}	0.16±0.12^{bcde}
6	3	1.45±0.08^{h}	0.168±0.015^{d}	3.60±0.80^{cde}	531.53±42.39^{fg}	0.14±0.03^{cde}
	4	1.57±0.08^{gh}	0.175±0.009^{d}	3.53±0.32^{de}	491.50±141.30^{g}	0.14±0.01^{de}
	5	1.34±0.20^{h}	0.224±0.089^{cd}	4.03±2.40^{bcde}	633.57±198.20^{efg}	0.216±0.01^{bcde}
	1	2.31±0.53^{abc}	0.283±0.083^{bcd}	4.93±3.07^{bcde}	1138.53±266.20^{a}	0.12±0.12^{bcde}
	2	1.82±0.48^{cdefgh}	0.341±0.048^{bcd}	4.53±2.58^{bcde}	929.20±92.30^{abcde}	0.18±0.10^{bcde}
9	3	1.70±0.39^{efgh}	0.234±0.052^{cd}	5.23±1.33^{bcde}	937.30±77.19^{abcde}	0.21±0.05^{bcde}
	4	1.95±0.25^{bcdefg}	0.327±0.102^{bcd}	4.83±0.67^{bcde}	752.20±119.14^{cdefg}	0.19±0.03^{bcde}
	5	1.81±0.12^{defgh}	0.350±0.175^{bcd}	5.33±2.10^{bcde}	881.13±193.43^{abcde}	0.21±0.08^{bcde}
	1	2.13±0.61^{bcdef}	0.300±0.032^{bcd}	2.63±1.10^{e}	823.33±166.60^{abcdefg}	0.11±0.04^{e}
	2	1.68±0.15^{fgh}	0.239±0.022^{cd}	6.10±1.31^{bcd}	753.77±344.19^{cdefg}	0.24±0.05^{bcd}
12	3	2.09±0.15^{bcdef}	0.254±0.071^{bcd}	5.00±2.15^{bcde}	937.43±195.11^{abcde}	0.20±0.09^{bcde}
	4	2.16±0.13^{bcdef}	0.343±0.100^{bcd}	5.17±1.37^{bcde}	885.20±175.17^{abcde}	0.21±0.05^{bcde}
	5	1.95±0.10^{bcdefg}	0.336±0.196^{bcd}	4.80±1.31^{bcde}	740.57±134.98^{cdefg}	0.19±0.01^{bcde}
	1	2.17±0.20^{abcdef}	0.251±0.175^{cd}	6.53±0.93^{bcd}	1072.00±31.29^{abc}	0.26±0.04^{bcd}
	2	1.95±0.25^{bcdefg}	0.240±0.174^{cd}	5.50±0.36^{bcde}	837.40±318.44^{abcdef}	0.22±0.01^{bcde}
15	3	2.06±0.25^{bcdefg}	0.232±0.116^{cd}	6.60±0.36^{bc}	973.03±135.72^{abcd}	0.26±0.01^{bc}
	4	2.33±0.18^{ab}	0.597±0.164^{a}	5.53±1.72^{bcde}	990.93±188.85^{abcd}	0.22±0.07^{bcde}
	5	2.39±0.36^{ab}	0.378±0.173^{bc}	9.83±1.46^{a}	1094.17±93.67^{ab}	0.39±0.06^{a}
	1	2.67±0.67^{a}	0.285±0.072^{bcd}	6.33±0.32^{bcd}	1133.33±439.23^{a}	0.25±0.01^{bcd}
	2	2.19±0.03^{abcde}	0.262±0.121^{bcd}	6.23±0.65^{bcd}	1107.03±107.63^{ab}	0.25±0.03^{bcd}
18	3	2.30±0.22^{abcd}	0.444±0.115^{ab}	6.83±0.32^{ab}	1009.30±90.95^{abcd}	0.27±0.01^{ab}
	4	2.27±0.31^{abcd}	0.229±0.089^{cd}	6.63±3.87^{bc}	964.77±484.63^{abcde}	0.27±0.15^{bc}
	5	2.28±0.23^{abcd}	0.304±0.099^{bcd}	4.83±0.40^{bcde}	967.70±11.07^{abcde}	0.19±0.02^{bcde}

Note: The data are presented as mean ± SD. Different letters (a-h) within the same column show significant differences among the samples (Fisher (LSD), $p < 0.05$.

OPTIMIZATION STUDY

Table S3. Swelling ratio over time of the bacterial cellulose membranes dependent on the incubation and inoculum volume.

Harvest day (d)	Inoculum volume (mL)	BC type	10 min	20 min	30 min	1 h	6 h	24 h
6	1	dry	879.4±83.9[abc]	1182.7±442.5[a]	1402.4±360.0[a]	1486.3±376.0[a]	1681.2±569.4[a]	1931.4±759.9[a]
18	1	dry	794.6±140.0[bc]	1008.9±130.2[a]	1189.4±274.6[a]	1310.2±287.4[a]	1500.0±420.4[a]	1924.0±530.5[a]
12	3	dry	694.8±66.5[c]	882.3±29.9[a]	1089.4±136.1[a]	1333.4±332.8[a]	1387.2±350.8[a]	1578.9±443.1[a]
6	5	dry	958.6±232.3[ab]	1290.0±587.6[a]	1374.5±665.7[a]	1653.8±951.3[a]	1814.0±953.4[a]	1900.4±900.4[a]
18	5	dry	1079.4±81.6[a]	1253.0±169.6[a]	1460.3±162.2[a]	1545.3±52.8[a]	1765.3±309.8[a]	2343.0±572.0[a]
6	1	moist	624.7±174.6[a]	809.6±98.2[a]	1187.7±476.5[a]	1321.3±536.8[a]	1428.4±585.9[a]	1481.8±567.4[a]
18	1	moist	887.7±1000.6[a]	1113.1±1121.9[a]	1282.3±1213.3[a]	1367.3±1439.9[a]	1435.2±1391.8[a]	1519.8±1421.4[a]
12	3	moist	628.9±105.6[a]	889.4±215.8[a]	1163.6±311.8[a]	1333.7±391.3[a]	1500.8±442.6[a]	1636.9±504.4[a]
6	5	moist	758.7±75.8[a]	1040.8±84.9[a]	1189.4±138.4[a]	1284.8±212.1[a]	1308.6±214.4[a]	1408.9±150.4[a]
18	5	moist	649.9±169.3[a]	1048.2±258.9[a]	1334.1±346.6[a]	1566.4±393.8[a]	1702.3±340.2[a]	1791.8±376.9[a]

Note: The data are presented as mean ± SD. Different letters (a-c) within the same column show significant differences among dry or moist BC samples (Fisher (LSD), $p < 0.05$)

Table S4. Moisture content over time of the bacterial cellulose membranes dependent on the incubation period and inoculum volume.

Harvest day (d)	Inoculum volume (mL)	BC type	10 min	20 min	30 min	1 h	6 h	24 h
6	1	dry	89.7±0.9[abc]	91.6±2.4[a]	93.1±1.5[a]	93.5±1.4[a]	94.0±1.6[a]	97.6±3.3[a]
18	1	dry	88.6±1.9[bc]	90.9±1.0[a]	92.0±1.5[a]	92.7±1.5[a]	93.5±1.5[a]	94.8±1.3[a]
12	3	dry	87.4±1.0[c]	89.8±0.3[a]	91.5±1.0[a]	92.7±1.8[a]	93.0±1.9[a]	93.7±1.9[a]
6	5	dry	90.2±2.3[ab]	92.0±2.9[a]	92.3±3.0[a]	93.1±3.3[a]	93.7±3.5[a]	94.2±2.7[a]
18	5	dry	91.5±0.6[a]	92.5±0.9[a]	93.5±0.7[a]	93.9±0.2[a]	94.5±0.9[a]	95.8±0.9[a]
6	1	moist	85.7±3.1[a]	88.9±98.2[a]	91.5±2.9[a]	92.2±3.0[a]	92.7±2.9[a]	93.0±2.8[a]
18	1	moist	82.1±11.7[a]	86.9±8.0[a]	89.0±6.4[a]	88.4±7.5[a]	89.8±6.1[a]	90.6±5.6[a]
12	3	moist	86.1±1.9[a]	89.6±2.0[a]	91.8±1.8[a]	92.7±1.8[a]	93.5±1.6[a]	93.9±1.7[a]
6	5	moist	88.3±1.1[a]	91.2±0.7[a]	92.2±0.9[a]	92.7±1.1[a]	92.8±1.1[a]	93.3±0.7[a]
18	5	moist	86.1±3.5[a]	91.0±2.2[a]	92.7±2.1[a]	93.7±1.7[a]	94.3±1.2[a]	94.6±1.2[a]

Note: The data are presented as mean ± SD. Different letters (a-c) within the same column show significant differences among dry or moist BC samples (Fisher (LSD), $p < 0.05$)

Table S5. Drug release over time of the bacterial cellulose membranes dependent on the incubation period and inoculum volume.

Harvest day (d)	Inoculum volume (mL)	BC type	30 min	1 h	2 h	3 h	6 h	24 h	48 h	72 h
6	1	dry	0.317±0.01^{a}	0.295±0.01^{ab}	0.237±0.01^{c}	0.262±0.01^{abc}	0.258±0.01^{bc}	0.256±0.01^{bc}	0.240±0.02^{bc}	0.217±0.02^{c}
18	1	dry	0.284±0.03^{a}	0.268±0.02^{ab}	0.218±0.03^{bc}	0.224±0.03^{bc}	0.233±0.03^{abc}	0.224±0.03^{bc}	0.218±0.02^{bc}	0.194±0.02^{c}
12	3	dry	0.248±0.05^{a}	0.244±0.04^{a}	0.211±0.04^{ab}	0.221±0.05^{ab}	0.211±0.05^{ab}	0.180±0.03^{bc}	0.152±0.03^{cd}	0.116±0.00^{d}
6	5	dry	0.489±0.07^{a}	0.484±0.08^{a}	0.438±0.06^{ab}	0.413±0.07^{abc}	0.371±0.05^{bc}	0.347±0.06^{bcd}	0.315±0.05^{cd}	0.251±0.04^{d}
18	5	dry	0.525±0.14^{a}	0.493±0.13^{a}	0.468±0.12^{a}	0.431±0.12^{ab}	0.394±0.08^{ab}	0.377±0.10^{ab}	0.343±0.08^{ab}	0.276±0.09^{b}
6	1	moist	1.385±0.04^{a}	1.293±0.06^{ab}	1.209±0.05^{bc}	1.164±0.04^{c}	1.102±0.04^{cd}	0.996±0.03^{d}	0.877±0.10^{e}	0.829±0.10^{e}
18	1	moist	1.445±0.31^{a}	1.343±0.28^{a}	1.242±0.26^{a}	1.182±0.26^{a}	1.099±0.24^{a}	1.042±0.23^{a}	1.016±0.28^{a}	0.985±0.31^{a}
12	3	moist	1.678±0.33^{a}	1.625±0.32^{a}	1.538±0.28^{ab}	1.454±0.26^{abc}	1.336±0.23^{abc}	1.099±0.29^{bc}	1.000±0.26^{c}	1.002±0.19^{c}
6	5	moist	1.043±0.20^{a}	0.936±0.15^{ab}	0.896±0.14^{ab}	0.825±0.15^{abc}	0.743±0.10^{bc}	0.745±0.15^{bc}	0.643±0.10^{c}	0.587±0.11^{c}
18	5	moist	1.330±0.18^{a}	1.290±0.16^{ab}	1.220±0.19^{ab}	1.100±0.18^{abc}	0.998±0.17^{abc}	0.957±0.20^{bc}	0.949±0.33^{bc}	0.759±0.17^{c}

Note: The data are presented as mean ± SD. Different letters (a-d) within the same column show significant differences among the samples (Fisher (LSD), $p < 0.05$)

Table S6. Characteristics of dry and moist bacterial cellulose membranes dependent on the harvest day and inoculum volume, mean±SD.

**BC type**	**Harvest (d)**	**Inoculum volume (mL)**	**Half moisture time (h)**	**Maximum load (N)**	**Elongation at break, ε (%)**	**Stiffness, k (kN/cm)**
dry	6	1	2.04±2.04^{a}	9.13±0.25^{ab}	6.95±1.79^{b}	25.79±6.08^{b}
		5	0.87±0.53^{ab}	8.50±3.34abc	4.92±0.46^{b}	28.80±7.30^{b}
	12	3	0.58±0.20^{b}	12.05±2.28^{a}	9.84±3.17^{b}	23.57±5.64^{b}
	18	1	1.06±0.69^{ab}	12.41±4.43^{a}	7.94±3.39^{b}	27.87±4.47^{b}
		5	1.23±0.57^{ab}	11.07±4.00^{a}	4.90±0.65^{b}	41.87±4.77^{a}
moist	6	1	0.42±0.11^{b}	3.63±0.77^{d}	18.77±1.04^{a}	3.20±0.59^{c}
		5	0.46±0.31^{b}	3.14±0.46^{d}	21.74±5.10^{a}	2.49±0.15^{c}
	12	3	0.47±0.08^{b}	3.49±0.99^{d}	16.11±1.36^{a}	4.32±2.38^{c}
	18	1	0.45±0.12^{b}	5.57±0.38^{bcd}	21.74±6.25^{a}	5.28±3.05^{c}
		5	0.33±0.03^{b}	4.80±0.66^{cd}	18.40±4.47^{a}	4.36±0.49^{c}

Note: The data are presented as mean ± SD. Different letters (a-d) within the same column show significant differences among the samples (Fisher (LSD), $p < 0.05$).
